# Environmental sustainability of biofuels: a review

**DOI:** 10.1098/rspa.2020.0351

**Published:** 2020-11-25

**Authors:** Harish K. Jeswani, Andrew Chilvers, Adisa Azapagic

**Affiliations:** 1 Department of Chemical Engineering and Analytical Science, The University of Manchester, Manchester M13 9PL, UK; 2 Royal Academy of Engineering, 3 Carlton House Terrace, London SW1Y 5DG, UK

**Keywords:** biofuels, carbon footprint, environmental impacts, life cycle assessment, transport, sustainability

## Abstract

Biofuels are being promoted as a low-carbon alternative to fossil fuels as they could help to reduce greenhouse gas (GHG) emissions and the related climate change impact from transport. However, there are also concerns that their wider deployment could lead to unintended environmental consequences. Numerous life cycle assessment (LCA) studies have considered the climate change and other environmental impacts of biofuels. However, their findings are often conflicting, with a wide variation in the estimates. Thus, the aim of this paper is to review and analyse the latest available evidence to provide a greater clarity and understanding of the environmental impacts of different liquid biofuels. It is evident from the review that the outcomes of LCA studies are highly situational and dependent on many factors, including the type of feedstock, production routes, data variations and methodological choices. Despite this, the existing evidence suggests that, if no land-use change (LUC) is involved, first-generation biofuels can—on average—have lower GHG emissions than fossil fuels, but the reductions for most feedstocks are insufficient to meet the GHG savings required by the EU Renewable Energy Directive (RED). However, second-generation biofuels have, in general, a greater potential to reduce the emissions, provided there is no LUC. Third-generation biofuels do not represent a feasible option at present state of development as their GHG emissions are higher than those from fossil fuels. As also discussed in the paper, several studies show that reductions in GHG emissions from biofuels are achieved at the expense of other impacts, such as acidification, eutrophication, water footprint and biodiversity loss. The paper also investigates the key methodological aspects and sources of uncertainty in the LCA of biofuels and provides recommendations to address these issues.

## 1. Introduction

Greenhouse gas (GHG) emissions from transport have been increasing at a faster rate than from any other sector [1]. The sector relies heavily on fossil fuels, which accounted for 96.3% of all transportation fuels in 2018 [2]. Transport is also responsible for 15% of the world's GHG emissions and 23% of total energy-related CO_2_ emissions [1]. To reduce dependence on petroleum-based fuels, as well as to mitigate climate change, biofuels are viewed widely as promising alternative transportation fuels.

Biofuels have been used since the early days of the automotive industry. For instance, Rudolph Diesel tested his first engine on peanut oil [3] after pulverized coal was found to be unsuitable. Until the 1940s, biofuels were seen as viable transport fuels and bioethanol blends, such as Agrol, Discol and Monopolin, were commonly used in the USA, Europe and other regions [3]. Further development of bioethanol ceased after the Second World War as petroleum-derived fuel became cheaper. During the oil crisis in the 1970s, many countries showed renewed interest in production of commercial biofuels; however, only Brazil started to produce ethanol at a large scale as part of the National Ethanol Programme ‘Proálcool’ [4]. During the late 1990s, with the rise in crude oil prices and concerns over energy security, the USA and many nations in Europe developed policies in support of domestic biofuel industries [5]. The interest in biofuels further increased in the past decade with the development of policies on climate change mitigation and strategies to reduce GHG emissions from the transport sector. More than 60 countries have since launched biofuel programmes and set targets for blending biofuels into their fuel pools [6]. The most notable are Renewable Fuel Standard (RFS) [7] in the USA and the Renewable Energy Directive (RED) in Europe [8].

Owing to these policies, world bioethanol production has increased by 67%, from 67 to 110.4 billion litres, over the decade of 2008–2018 [2]. During the same period, biodiesel production increased more than threefold, from 12 to 41 billion litres. Currently, biofuels account for about 3.4% of total transportation fuels worldwide [2]. The global production of biofuels is dominated by the USA and Brazil—producing 69% of all biofuels in 2018—followed by Europe (EU-28) with 9% [9]. Production of bioethanol in the USA is almost exclu­sively from corn, whereas in Brazil, it is from sugarcane. In Europe, the main feedstocks are corn, wheat and sugar beet for bioethanol, while rapeseed and used cooking oil (UCO) are used for biodiesel production [10]. Argentina, Brazil and the USA also produce significant quantities of biodiesel, predominantly from soya bean, while Malaysia and Indonesia produce biodiesel from palm oil. Several international and national organizations have made mid- and long-term projections for global production of biofuels. These projections provide wide-ranging estimates of potential future increases in liquid biofuels for transport globally. The International Energy Agency (IEA) estimates that as much as one-third of all transportation fuel could come from biofuels by 2050 [11], while organizations, such as the OECD and BP, project approximately a 7% share of biofuels by 2030 [12]. A recent assessment [13] also suggests that the IEA projections could be impossible to achieve, estimating the maximum potential of transport biofuels by 2050 to be at least 30% lower than those projected by the IEA.

**Figure 1.**
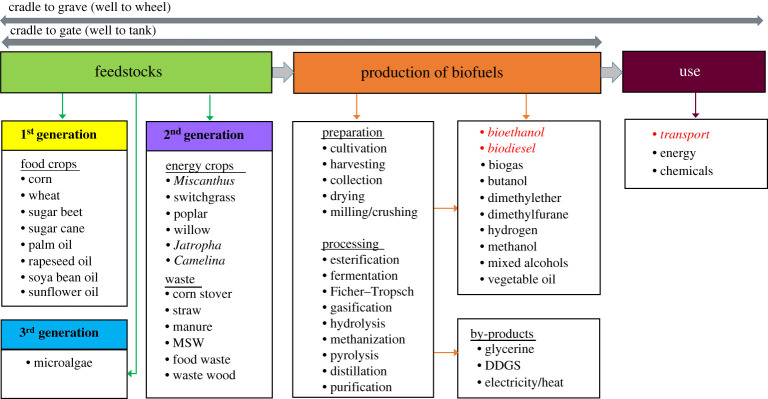
An overview of feedstocks and production processes for different biofuels, also showing the life cycle of fuels from cradle to gate (well to tank) and cradle to grave (well to wheel). Adapted from [14]. The figure has been simplified and other feedstocks, production routes, products/by-products and uses are possible. The italic font denotes the focus of this review, i.e. bioethanol and biodiesel used for transportation. DDGS, dark distillers grain with solids. (Online version in colour.)

Biofuels can be differentiated according to a number of key characteristics, including feedstock type, conversion process, technical specification of the fuel and its use. Owing to this multitude of possible distinctions, various definitions are in use for biofuel types. Two commonly used typologies are ‘first, second and third generation’ and ‘conventional and advanced’ biofuels. Biofuels produced from food or animal feed crops are referred to as first-generation biofuels. Since first-generation biofuels are produced through well-established technologies and processes, such as fermentation, distillation and transesterification, they are also commonly referred to as ‘conventional biofuels'. A key characteristic for second-generation biofuels is that they are derived from non-food feedstocks, such as dedicated energy crops (e.g. *Miscanthus*, switchgrass, short rotation coppice (SRC) and other lignocellulosic plants), agricultural residues, forest residues and other waste materials (e.g. UCO and municipal solid waste). Biodiesel produced from microalgae through conventional transesterification or hydro-treatment of algal oil is commonly known as third-generation biofuel. Second- and third-generation biofuels are often referred to as ‘advanced biofuels’ as their production techniques or pathways are still in the research and development, pilot or demonstration phase. In this paper, the terminology ‘first, second and third generation’ has been selected and followed throughout. An overview of different biofuel types, their feedstocks and conversion routes can be seen in figure 1.

Biofuels offer both advantages and disadvantages in terms of environmental, economic and social sustainability [14]. On the one hand, reduction in GHG emissions, energy security and rural development are the most important drivers for biofuels globally. On the other hand, there are concerns related to increasing the production of biofuels, such as upward pressure on food prices, the risk of increase in GHG emissions through direct and indirect land-use change (LUC) from production of biofuel feedstocks, as well as the risks of degradation of land, forests, water resources and ecosystems [15]. The use of first-generation feedstocks, such as corn, has become a particularly contentious issue, largely owing to competition with food production and concerns over diverting agricultural land into fuel production. A growing demand for agricultural produce risks an increase in deforestation and use of land with a high biodiversity value to meet this demand, as well as associated usage of freshwater, fertilizers and pesticides, with negative consequences on the environment. Some of these issues could be addressed by using second-generation feedstocks; however, the economic viability of some second generation of biofuels remains doubtful in the current economic context, largely because of the low oil prices [16–18]. Third-generation (algal) biofuels could also avoid the issue of food competition and land use because microalgae can be grown on non-arable land and in wastewater, saline or brackish water and they grow extremely rapidly. However, the production of biofuels from microalgae is energy-intensive and at present economically unviable [19].

To encourage sustainable development of biofuels, regulatory policies, such as the RED and RFS, stipulate various sustainability criteria for biofuels. One of the main criteria is related to life cycle GHG emissions. The RED stipulates that biofuels should have at least 50% lower emissions than their fossil fuel alternatives for installations in operation before October 2015 and 60% for installations starting after this date, rising to 65% lower for biofuel plants commencing operation after 1 January 2021 [8]. RFS requires producers of advanced biofuels to reduce GHG emissions by at least 50%, while standard biofuels have to achieve a 20% reduction in GHG emissions [7]. The climate change impact related to GHG emissions and other sustainability aspects of biofuels should be evaluated on a life cycle basis via life cycle assessment (LCA) to avoid shifting burdens from one part of the life cycle or supply chain to another.

Numerous LCA studies have considered the potential of biofuels to achieve reductions in life cycle GHG emissions by estimating their potential impact on climate change. However, their findings are often conflicting, with a wide variation in the estimates. A number of review papers have also discussed LCA of biofuels, but these focused on a particular aspect, such as a region, feedstock or type of biofuel. For example, Shonnard *et al*. [20] reviewed LCA studies of biofuels in the Pan American region. Morales *et al*. [21] and Roy *et al*. [22] concentrated on LCA studies of lignocellulosic bioethanol, while Menten *et al*. [23] focused on advanced biofuels. Sieverding *et al*. [24] conducted a review of soya bean-based biodiesel and van Eijck *et al*. [25] reviewed issues pertaining to biodiesel from *Jatropha*.

However, a comprehensive review of all types of biofuels is not available in the literature. Besides, many more LCA studies on biofuels have been published since the publication of the above-mentioned reviews. Thus, this review paper aims to close this gap by analysing and synthesizing the latest information concerning LCA of biofuels. The main objective is to provide a greater clarity and understanding of the environmental sustainability of different liquid biofuels with the aim of informing future policy. A further objective is to examine the state-of-the-art knowledge on environmental issues associated with the production and consumption of biofuels. The next section provides an overview of the reviewed LCA studies in terms of their coverage with regard to biofuel type, geographical location and their approaches to handling critical methodological aspects in LCA. Section 3 presents results for the climate change impact, energy and water use and other environmental issues associated with different types of biofuels. The key methodological aspects and sources of uncertainty in assessing the environmental impacts of biofuels are investigated and discussed in detail in §4. The paper ends with conclusions and recommendations on addressing the key issues related to sustainability of biofuels.

## 2. Study methodology and coverage

A systematic literature search was performed in different databases (Science Direct, Web of Science, Scopus and relevant academic journals) to identify academic, peer-reviewed studies on the environmental sustainability of biofuels. To avoid outdated information, the review of the literature predominantly focused on the articles published in the period from 2009 to 2020. Some important earlier publications cited frequently in the literature were also taken into account. In total, 179 articles were primary (original) LCA studies, combining between them 613 assessments of different types of biofuels, all of which are included in this review. In addition, further publications focusing on environmental issues not usually included in LCA studies, such as water footprint, biodiversity and LUC, as well as discussing various methodological aspects were also reviewed. These studies covered a wide spectrum of first-, second- and third-generation biofuels produced from more than 20 types of feedstock. Table 1 provides an overview of the LCA studies related to the types of fuel, feedstock and geographical coverage. Among them, 52% assessed first generation, 38% considered second generation and the remaining 10% assessed third-generation biofuels. Regarding the type of biofuel, 56% of studies were for bioethanol and the rest for biodiesel. Geographically, 36% of studies were based in Europe, 26% in North America, 20% in Asia, 12% in South America, 4% in Africa and 1% in Australia. An overview of how different studies approached some critical LCA methodological aspects, including type of LCA, goal and scope of the study, definition of the functional unit, allocation methods and estimation of the impacts, is provided below.

**Table 1.** An overview of the number of LCA studies by biofuel type, feedstock, location and land-use change.

	location						land-use change		
fuel type/feedstock	Europe	North America	South America	Asia	Africa	Australia	without	with	total^a^
*bioethanol—1st gen.*									
corn	6	23	0	1	0	0	16	14	30
molasses	4	12	0	25	3	4	30	18	48
sugar beet	19	1	0	0	1	0	14	7	21
sugarcane	0	4	32	1	1	0	28	10	38
wheat	39	0	0	0	0	0	28	11	39
*bioethanol—2nd gen.*									
bagasse	1	1	3	1	0	0	6	0	6
forest residue	16	7	0	0	0	0	23	0	23
*Miscanthus*	14	9	0	0	0	0	16	7	23
short rotation coppice	29	2	0	0	0	0	17	14	31
stover	12	18	0	0	0	0	27	3	30
straw/husk	27	1	0	9	0	0	32	5	37
switchgrass	2	17	1	0	0	0	18	2	20
*biodiesel—1st gen.*									
palm oil	0	0	3	56	0	0	32	27	59
rapeseed	19	13	2	0	4	0	24	14	38
soya bean	3	10	18	5	3	0	29	10	39
sunflower	1	0	2	0	5	0	5	3	8
*biodiesel—2nd gen.*									
*Camelina*	1	13	0	0	0	0	14	0	14
*Jatropha*	0	0	7	8	7	0	18	4	22
used cooking oil/tallow	17	1	3	5	1	0	27	0	27
*biodiesel—3rd gen.*									
algae	13	28	4	13	0	2	60	0	60
total	223	160	75	124	25	6	464	149	613

^a^The total number of studies or analyses, rather than the number of papers published, as some papers included several studies or analyses.

### (a) Type of life cycle assessment: attributional versus consequential

Two general types of LCA studies are distinguished: attributional (ALCA) and consequential (CLCA). They address different questions and follow different methodologies, and will normally have very different results. ALCA accounts for impacts directly related to the system of interest, attributing them to the activities within the system; hence, the term ‘attributional’. For biofuels, it is used mainly as an ‘accounting’ tool for estimating environmental impacts of various activities in the supply chain, comparisons of alternative systems and identification of environmental hotspots that can be targeted for improvements. CLCA, in addition to direct, also examines potential indirect consequences of the system under study by considering various ‘what if’ scenarios that could arise owing to this system; examples include changes in demand for the product of interest or technological improvements. For instance, CLCA can consider potential impacts of biofuel feedstock cultivation on other land-using sectors and the effect this might have on the food production system and LUC elsewhere in the global economy [26,27]. As such, CLCA is more suited for policy applications.

CLCA is still under development and, consequently, most of the LCA studies on biofuels found in the literature are attributional. Nevertheless, both the ALCA and CLCA are considered in this review.

### (b) Goal and scope of studies

Goal and scope definition is an important initial step in LCA studies as the specific methodological approaches depend strongly on the specific goal, scope and question being addressed. The goal and scope of the study influence the definition of the system boundary and determine what activities and life cycle stages will be considered [28]. LCA studies of biofuels have addressed a wide range of goals and research questions, including:

— What are the environmental impacts of the biofuel system under examination?
— How do biofuels compare with fossil fuels?
— What are the environmental hotspots in the life cycle of particular biofuel systems under study?
— What are the improvement options to optimize the supply chain under study?
— What are the environmental implications of biofuel policies?

Although the ISO 14040 LCA standard [28] requires clear definition of the goal and scope, a lack of or unclear definition of goal and scope is a common problem in LCA studies of biofuels. This can also mean that the study method and rationale can be unclear, making comparability of results difficult [29].

Two types of system boundaries were used in the reviewed LCA studies of biofuels: ‘cradle to gate’ (or ‘well to tank’) and ‘cradle to grave’ (or ‘well to wheel’); figure 1. However, the latter system boundary is more appropriate as it is important to include the use of fuels to enable comparisons of biofuels with their fossil substitutes, since the combustion performance and associated emissions of biofuels can significantly differ from their fossil substitutes for the same type of vehicle [30,31]. Around half (48%) of the LCA studies reviewed considered a cradle to grave system boundary to compare environmental impacts of biofuels with fossil fuels, while the rest were from cradle to gate. Other inconsistencies include the omission in some studies of various inputs (such as enzymes, pesticides, fertilizers, etc.) and co-products. These differences are often important enough to influence the results significantly.

### (c) Functional unit

In LCA, the term ‘functional unit’ describes the function of the system under study and represents the unit of analysis on which the study is based. The choice of the functional unit is driven by the goal of the study and must be representative of the system(s) studied and their main purpose (function). Biofuels regulations, such as the RED [8] and RFS [7], use the energy content of biofuels (MJ) as the functional unit. While this functional unit was often used in the reviewed literature, others include the distance travelled by a vehicle (vehicle^.^km) [21,32], volume (litre) [33,34] and mass (kilogram or tonne) [17,35] of biofuels. Some studies also used the mass of biofuel feedstock [36,37], agricultural land area [38,39] and annual operation of refinery [40]. The use of such a wide array of functional units makes comparisons of LCA studies challenging.

### (d) Allocation methods

Biofuel production processes often produce several co-products, such as animal feed, heat, electricity and biochemicals. Therefore, to determine the impacts from the biofuel of interest, it is necessary to allocate the impacts between the biofuel and its co-products. The ISO 14040 and 14044 standards recommend that, if possible, allocation should be avoided through subdivision of processes, or by system expansion. The latter involves expanding the system boundary to include alternative ways of producing the co-products. The production system is then credited for displacing production of the co-products in the alternative systems by subtracting their impacts from the impacts of the biofuel production system. Hence, this method is also known as ‘substitution’ or the ‘avoided burden’ approach. If allocation cannot be avoided, the impacts can be apportioned between the biofuel and the co-products using allocation factors based on physical or economic relationships. Mass and energy content of biofuels and co-products are often used to derive allocation factors based on physical relationships. Economic allocation is based on the assumptions that the market prices are the driver for the production process and the impacts are apportioned in proportion to the economic value (cost or price) of the biofuel and the co-products. In LCA of biofuels, the most common approaches used to allocate the impacts are system expansion and allocation by the energy content. This perhaps reflects the regulatory requirements in the USA and Europe: RFS [7] prefers system expansion, while the RED [8] favours allocation based on the energy content of biofuels.

### (e) Land-use change

LUC is an important source of GHG emissions that contributed 660 ± 290 Gt CO_2_ to the atmospheric CO_2_ in the period from 1750 and 2011 [41]. The majority of LUC is driven by demand for food, fibre and fuel [42]. Converting natural vegetation or forest to cultivate biofuel feedstocks releases a significant amount of carbon from soil and plant biomass, creating a ‘carbon debt’ that can take years to repay [43,44]. Furthermore, cultivation of biofuel feedstocks on land that has high soil carbon content, such as peat land, leads to a considerable increase in GHG emissions [45]. Besides increasing GHG emissions, changes in land use can have other environmental consequences, such as soil erosion, nutrient depletion, water consumption and loss of biodiversity [46]. LUC related to biofuels can occur in two ways: direct (DLUC) or indirect (ILUC). DLUC refers to the direct transformation of previously uncultivated areas (such as grasslands and forests) into croplands for biofuel feedstock production. ILUC occurs when additional demand for biofuel feedstock induces displacement of food and feed crop production to new land areas previously not used for cultivation. Only 25% of the reviewed LCA studies took LUC into account.

### (f) Environmental impacts

GHG emissions and savings in comparison to fossil fuels are the centre of attention in most LCA studies on biofuels. Other environmental impact categories considered in biofuel LCA studies include acidification, eutrophication, photochemical smog, human toxicity and eco-toxicity. However, the number of studies that have assessed a wider set of impact categories is still limited: of the 179 (primary) LCA studies reviewed, only 40% of such studies were found in the literature. These are discussed in the next section, starting with the climate change impact, or global warming potential (GWP) as often referred to in LCA, and following on to discuss energy and water use, biodiversity and other impacts.

## 3. Environmental impacts of biofuels

### (a) Global warming potential

**Figure 2.**
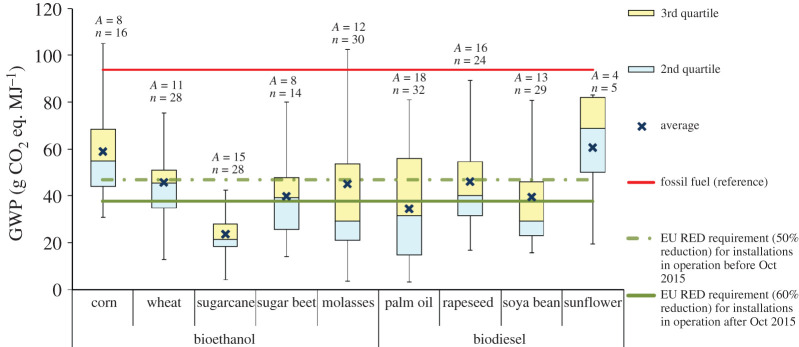
GWP of first-generation biofuels without land-use change. Based on data from [24,32,34,47–118]. For the box plot legend, see electronic supplementary material, figure S1 and for the data used to plot this graph, see electronic supplementary material, figure S2. ‘Fossil fuel (reference)’ is the average carbon intensity of petrol and diesel supplied in the EU (94 g CO_2_ eq. MJ^−1^) as specified in the RED [8]. ‘*A*’ refers to the number of LCA articles found in the literature and ‘*n*’ denotes the total number of analyses. (Online version in colour.)

**Figure 3.**
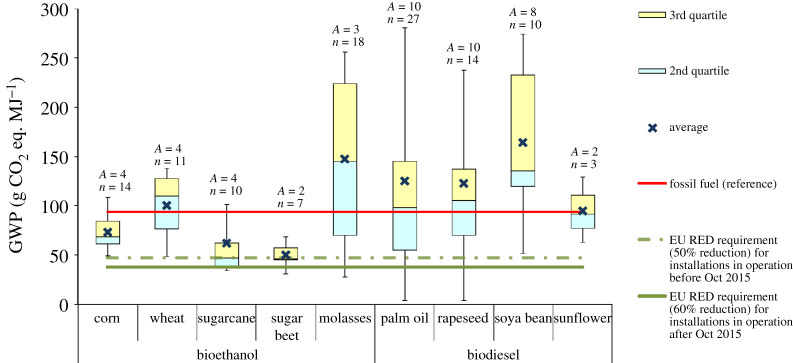
GWP of first-generation biofuels with land-use change. Based on data from [32,35,48,51,56,58,62–64,66–72,79,86,87,91,108,116,119–127]. For the box plot legend, see electronic supplementary material, figure S1 and for the data used to plot this graph, see electronic supplementary material, figure S3. ‘Fossil fuel (reference)’ is the average carbon intensity of petrol and diesel supplied in the EU (94 g CO_2_ eq. MJ^−1^) as specified in the RED [8]. ‘*A*’ refers to the number of LCA articles found in the literature and ‘*n*’ denotes the total number of analyses. (Online version in colour.)

**Figure 4.**
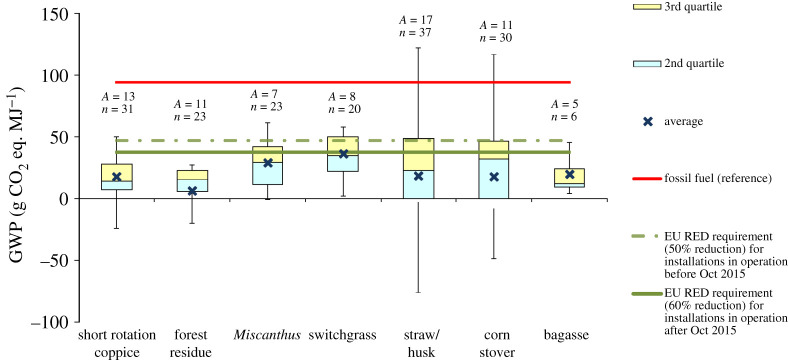
GWP of second-generation bioethanol. Based on data from [17,18,33,34,40,66,68,119,120,102,104,105,115,117,128–163]. The negative values are due to the credits for co-products, such as heat and chemicals. For the box plot legend, see electronic supplementary material, figure S1 and for the data used to plot this graph, see electronic supplementary material, figure S4. ‘Fossil fuel (reference)’ is the average carbon intensity of petrol and diesel supplied in the EU (94 g CO_2_ eq. MJ^−1^) as specified in the RED [8]. ‘*A*’ refers to the number of LCA articles found in the literature and ‘*n*’ denotes the total number of analyses. (Online version in colour.)

**Figure 5.**
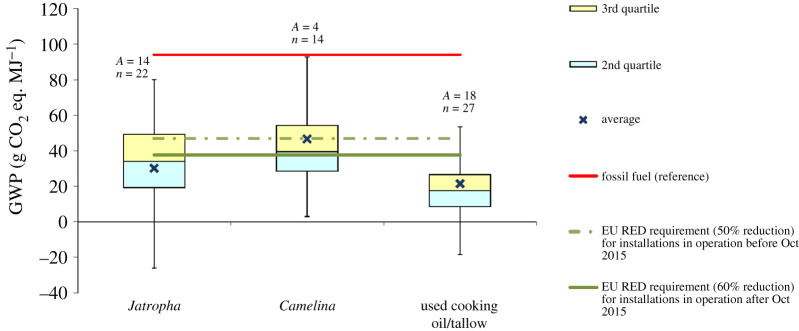
GWP of second-generation biodiesel. Based on data from [20,47,89,90,97,108,164–189]. The negative values are due to the credits for co-products. For the box plot legend, see electronic supplementary material, figure S1 and for the data used to plot this graph, see electronic supplementary material, figure S5. ‘Fossil fuel (reference)’ is the average carbon intensity of petrol and diesel supplied in the EU (94 g CO_2_ eq. MJ^−1^) as specified in the RED [8]. ‘*A*’ refers to the number of LCA articles found in the literature and ‘*n*’ denotes the total number of analyses. (Online version in colour.)

**Figure 6.**
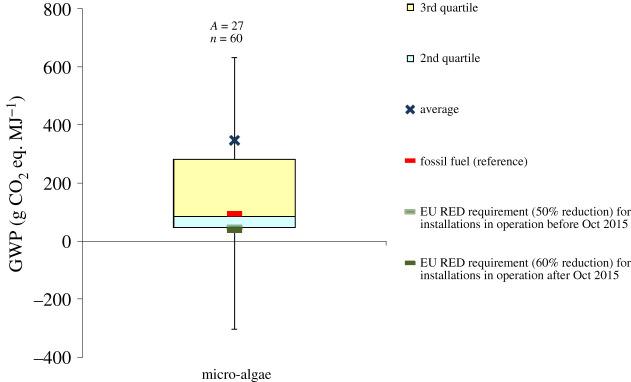
GWP of microalgae biodiesel. Based on data from [19,85,97,113,141,167,190–209]. The negative values are due to the credits for co-products and avoided processes, such as wastewater treatment. For the box plot legend, see electronic supplementary material, figure S1 and for the data used to plot this graph, see electronic supplementary material, figure S6. ‘Fossil fuel (reference)’ is the average carbon intensity of petrol and diesel supplied in the EU (94 g CO_2_ eq. MJ^−1^) as specified in the RED [8]. ‘*A*’ refers to the number of LCA articles found in the literature and ‘*n*’ denotes the total number of analyses. (Online version in colour.)

For this impact, the LCA studies present contradictory results, ranging from favourable to unfavourable, even for the same type of feedstock. This is a result of the differences in the assumptions, data sources, allocation methods and LUC. The influence of these aspects is discussed in detail in §4. The GWP of biofuels reported in the reviewed LCA studies is summarized in figures 2–6 and discussed below for different types of biofuel. For further details, see electronic supplementary material, figures S1–S7.

#### (i) First-generation biofuels

As first-generation biofuels may involve LUC, which in turn can affect significantly the total GWP, the results reported in the literature are discussed first for the cases without and then with LUC.

*GWP without land-use change*. As can be observed in figure 2, the GWP of first-generation bioethanol from different food crops vary considerably, ranging from 3 to 162 g CO_2_ eq. MJ^−1^ (see electronic supplementary material, figure S2). Figure 2 also shows that the average GWP of bioethanol is lower than that of petrol for all the feedstocks (23–59 versus 94 g CO_2_ eq. MJ^−1^). However, only bioethanol from sugarcane can meet the RED requirement of 60% reduction in GHG emissions relative to petrol. The average reductions in emissions from the other four feedstocks—corn, wheat, molasses and sugar beet—are not sufficient to meet this requirement. The main reasons that bioethanol from sugarcane can meet the 60% reduction requirement are relatively lower inputs of agro-chemicals and higher yields of sugarcane crops as well as the credits for electricity produced as a co-product in a biorefinery.

The GHG emissions for first-generation biodiesel also show a large variation across the LCA studies, with the GWP ranging between 3 and 111 g CO_2_ eq. MJ^−1^ (electronic supplementary material, figure S2). However, as shown in figure 2, the average GWP of biodiesel from all the feedstocks considered is lower than that of fossil diesel. Nevertheless, only biodiesel from palm oil meets the RED requirement for 60% reduction of the GWP relative to diesel (average value). Rapeseed and soya bean also come close to fulfilling this requirement, but sunflower biodiesel cannot meet even the 35% reduction.

The large variability in the GWP of first-generation biofuels shown in figure 2 is due to several reasons. For example, the LCA study on corn ethanol and soya bean biodiesel production in China found that the GWP of corn ethanol and soya bean biodiesel were 40 and 20% higher than petrol and diesel, respectively, owing to the relatively higher use of fertilizers, higher process energy consumption and the coal-dominated energy mix of China [47]. Low or no GHG savings (0–20%) compared to the fossil fuels were reported for South African sugar beet bioethanol as well as rapeseed, soya bean and sunflower biodiesel due to water scarcity affecting crop yields [48,49]. On the other hand, owing to higher yield and lower farming inputs, soya bean biodiesel produced in Brazil, the USA and Argentina achieve more than 60% GHG savings relative to the fossil fuels [50]. Studies on palm [51,52], rapeseed [53,54] and sunflower [55] biodiesel have also found the significant effect on GHG emissions of different locations, farming practices and usage of fertilizers. In the case of palm oil, the emissions vary with the options for handling of methane emissions from the treatment of palm oil milling effluent [56]. The influence of the fuel used in the biorefinery was noted in the case of wheat ethanol, with the GHG savings varying from 4 to 85% depending on whether straw or distillers' dried grains are used as a fuel [57]. Similarly, another study on molasses-based ethanol found that the use of bagasse instead of fuel oil would reduce GHG emissions of bioethanol from 112 to 51 g CO_2_ eq. MJ^−1^ [58]. A number of studies on biofuels from different feedstocks have also found that the emissions are highly influenced by the utilization of by-products and the allocation method [52,59–62]. The effects of allocation methods and other factors are discussed in more detail in §4.

*GWP with land-use change*. As shown in figure 3, if LUC is involved and considering the average GWP values, bioethanol cannot meet the 60% GHG reduction requirement regardless of the type of feedstock [62,63,119,120]. The increasing demand for bioethanol from sugarcane in Brazil has led to a continuous expansion of land used for sugarcane cultivation [44,210]. If this involves deforestation of tropical rainforest, the GWP of bioethanol from sugarcane can be up to 60% higher than that of petrol [58]. Similarly, expansion of soya bean cultivation in Central and South America is driving both direct and indirect LUC [121,211]. Furthermore, palm cultivation in Malaysia and Indonesia is associated with deforestation and drainage of peat lands. As a consequence, biodiesel from palm oil on peat and forest lands can have 3–40 times higher GHG emissions than diesel [64]. A recent study assessing the LUC impact of biofuels consumed in Europe [212] also found that the GWP of palm oil and soya bean diesel are almost two times higher than that of diesel. The same study also estimated the GWP of biodiesel from rapeseed and sunflower to be 20 to 40% higher than from conventional diesel (figure 3).

The significant variability shown in figure 3 for the GWP related to LUC is due to several reasons. For instance, some studies considered only ILUC [62,63,119,120] or DLUC [58,65–67], while others included both [68,122]. Furthermore, some studies applied partial equilibrium models and counterfactual (what if) scenarios to estimate ILUC emissions [62,119], whereas others used ILUC factors recommended by the US EPA [120]. The former tended to obtain higher ILUC emissions (34–155 g CO_2_ eq. MJ^−1^) [62,119,123] than the latter (5–16 g CO_2_ eq. MJ^−1^) [63,68,122]. For DLUC, several studies focused only on soil organic carbon (SOC) changes [65,122], but others also considered changes in the carbon stock related to the removal of biomass, both above and below the ground [58,67,68]. DLUC emissions also depend on the type of converted land and its previous use. For example, studies assuming palm oil cultivation on tropical forest and/or peat land in Malaysia and Indonesia estimated DLUC emissions in the range of 150–530 g CO_2_ eq. MJ^−1^ [64,69,70,124]. On the other hand, studies on palm oil in Colombia and Thailand considered increase in the carbon stock due to LUC, assuming that the expansion of oil palm cultivation would occur in shrublands, savannahs, paddy fields and other agricultural lands [52,56,71]. In the case of sugarcane, molasses and soy, LUC emission reported in the literature varied from 30 to 200 g CO_2_ eq. MJ^−1^ depending on the previous land use [35,58,69,72,121].

#### (ii) Second-generation biofuels

Figures 4 and 5 indicate that in most of the studies, the GWP of second-generation biofuels is considerably lower than that of fossil fuels. However, there is a large variation among different studies and feedstocks, with the values ranging from −115 to 173 g CO_2_ eq. MJ^−1^ for bioethanol and −88 to 150 g CO_2_ eq. MJ^−1^ for biodiesel (see electronic supplementary material, figures S4 and S5). These variations reflect the diversity of feedstocks and production routes, technology assumptions and methodological differences. Furthermore, some studies also considered emissions from ILUC [119] and SOC sequestration [68,128] associated with the production of SRC and perennial grasses as well as the reductions in SOC with removal of agricultural residues used as biofuel feedstocks [68,129]; for further discussion on SOC, see §4f. It should also be noted that the uncertainties related to technologies plays a particularly important role in the assessment of advanced biofuels as these are yet to be fully commercialized. Therefore, the quality of the available data is not as robust as in the case of the well-established first-generation biofuels.

In general, lignocellulosic bioethanol from agricultural and forest residues has a lower GWP than bioethanol from energy crops ( figure 4). This is largely due to N_2_O emitted during the cultivation of energy crops, related to the use of fertilizers. The latter are avoided in the case of residues as they are assumed to have no environmental burdens, which are all allocated to the original crop from which the waste is derived. In lignocellulosic bioethanol studies, the residual lignin is assumed to co-generate heat and power to meet the energy needs of the process, with surplus electricity exported to the grid. The biofuel production system is thus credited for avoiding the GHG emissions from the equivalent amount of grid electricity. For some feedstocks (SRC, forest residue, straw and corn stover), the credits for electricity generation and other co-products are higher than the total emissions from the biofuel production. Consequently, these studies report negative GWP, indicating the avoidance (saving) of GHG emissions. Some studies on energy crops also considered the increase in the carbon stock on the land that was converted to produce these crops, which led to the total net-negative GHG emissions [68,119]. On the other hand, harvesting of agricultural and forest residues can result in reduction of the land carbon stock [213–215], thus increasing GHG emissions [214,215]; however, most of the studies did not account for these changes. In the case of bioethanol from agricultural residues, other factors, such as the consideration of agricultural emissions [115], pre-treatment methods [130] and source of energy for the biorefinery [33,131], have a significant influence on GHG emissions.

While the LCA literature on second-generation bioethanol covers a wide range of feedstocks, the studies of biodiesel are more limited, focusing largely on three feedstocks: *Jatropha, Camelina* and UCO/tallow. As can be seen in figure 5, the GWP of *Jatropha* and *Camelina* varies widely because of variations in the yield in different regions and differences in processes and assumptions, especially with respect to co-product allocation. For example, the yield of *Jatropha* oil seeds varies in different studies by a factor of 30, from 0.4 to 12 t ha^−1 ^yr^−1^ [25]. The influence of allocation is also significant: using system expansion according to the US EPA methodology results in the GWP of *Jatropha* biodiesel of −88 g CO_2_ eq. MJ^−1^, while energy allocation as per the RED approach leads to GHG emissions of 15–20 g CO_2_ eq. MJ^−1^ [20].

Although a majority of the studies of biodiesel from UCO report that GHG savings are greater than the RED reduction target of 60%, some studies also estimate that the GHG savings from this type of biodiesel are not sufficient to meet the target (figure 5). This is due to some specific assumptions. For example, Intarapong *et al*. [90] considered pyrolysis for conversion of UCO to biodiesel, which is more energy-intensive than transesterification. Similarly, another study [47] assumed only a 5% biodiesel production yield, which is very low compared to more than 90% considered in other studies. Furthermore, Escobar *et al*. [171], who used consequential LCA methodology, considered indirect impacts, such as changes in the production of palm oil, soya bean and animal feed, found that the GWP of UCO biodiesel would be only 25% lower than that of diesel if ILUC and other indirect market impacts are considered.

#### (iii) Third-generation biofuels

In total, 27 LCA studies have estimated the GWP of third-generation algal biodiesel. However, they have all used very different approaches, process designs, system boundaries, methodologies and assumptions for feedstocks, nutrients and co-product management. As a result of the variation in these choices, the GWP differs widely between the studies, ranging from −2400 to 2880 g CO_2_ eq. MJ^−1^ (figure 6; electronic supplementary material, figure S6). These results would suggest that microalgae diesel can either reduce or increase GHG emissions significantly, relative to diesel, depending on the assumptions. However, a majority of the studies conclude that, at present state of development, algal biodiesel has higher life cycle GHG emissions than that of fossil diesel. The main reasons for higher emissions include lower algal yield [19,190] and high energy use in the cultivation, harvesting and drying stages [191–193].

Some studies which reported the high savings of GHG in comparison to diesel are based on the best-case assumptions that may not be feasible for large-scale implementation. These include the use of CO_2_ from cement plants as a feedstock [216], cane sugar as a nutrient/feedstock [217] and recycling of nutrients from anaerobic digestion plants [194] or wastewater [190].

### (b) Energy use

Various indicators have been used in LCA studies to quantify energy use in the life cycle of biofuels, including fossil energy consumption, primary, secondary or cumulative energy demand and net energy ratio [218]. However, many focused on fossil energy consumption, given that energy security and reducing dependence on fossil fuels are key objectives of national policies on biofuels, in addition to climate change mitigation.

**Figure 7.**
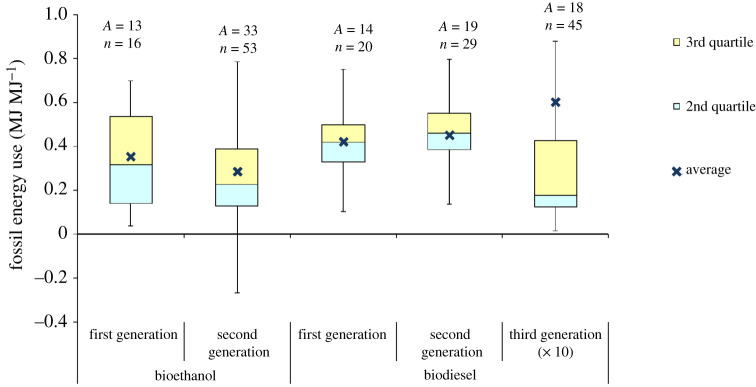
Fossil energy use in the life cycle of biofuels. Based on data from [17–19,40,47,48,54,55,57,58,60,68,76,77,80,85,89,92,93,95,96,98,108,113–115,120,121,129,132,133,136,138,139,143,144,147,152–154,156,159,161,162,164–167,169–171,175,176, 179–181,187,190–192,195–199,204,206,208,219–223]. For the box plot legend, see electronic supplementary material, figure S1 and for the data used to plot this graph, see electronic supplementary material, figure S7. The value for third-generation biodiesel should be multiplied by 10 to obtain the actual value. ‘*A*’ refers to the number of LCA articles found in the literature and ‘*n*’ denotes the total number of analyses. (Online version in colour.)

As indicated in figure 7, most of the studies estimate that the fossil energy consumption for first- and second-generation biofuels is below 0.5 MJ MJ^−1^. However, there is a wide variation across different types of biofuel, ranging from 0.04 to 0.86 MJ MJ^−1^ for first generation and from −0.57 to 0.87 MJ MJ^−1^ for second-generation biofuels (see electronic supplementary material, figure S7), where negative values are due to energy credits for co-products, such as electricity and heat. These variations are due to several factors, including differences in feedstock productivity, agricultural practices, conversion technologies and allocation methods. The results are also affected by the assumption on the type of energy (biomass or fossil) used in the conversion process.

The range of estimates for fossil fuel demand in the life cycle of algal biodiesel is even wider, ranging from 0.15 to 40.5 MJ MJ^−1^ (figure 7; electronic supplementary material, figure S7). Like the GWP, the reasons for these variations are technological uncertainties and the diversity of potential feedstocks and production systems. However, most studies agree that algal biofuels are not energetically viable because of high energy requirements for pumping, dewatering, lipid extraction and thermal drying [141,191,224–226]. In general, algae cultivation in raceway ponds has lower energy demand than photo-bioreactors, with some studies suggesting that the former can have energy demand below 1 MJ MJ^−1^ [225].

### (c) Water use

Water use in the production of feedstocks can be high, particularly for first-generation biofuels [5,227,228]. This is of concern where requirements for irrigation water for certain feedstocks might compete with water used for other purposes, such as food production. With increased agricultural biomass production for biofuels, the total global water consumption could increase significantly by 2050 [229] and, in areas that are already water stressed, additional water demand has a potential to substantially increase the overall environmental impacts of biofuels.

Water use is usually not included in LCA studies of biofuels, but there are numerous studies that have specifically focused on this aspect of biofuels production. Most of these provide a volumetric usage of water, such as the amount of green (soil moisture) and blue (surface) water. This is not sufficient to assess local environmental impacts of water consumption as these are highly dependent on the level of water availability in the local area and the specific characteristics of the hydrological cycle, even if the quantity used is the same for a particular product [230]. Furthermore, consideration of green water results in very large total water use for most agricultural crops. Since the local hydrological cycle may in reality be affected little by the use of green water in agriculture, inclusion of green water could overestimate the actual impact of water use for biofuels [231].

**Figure 8.**
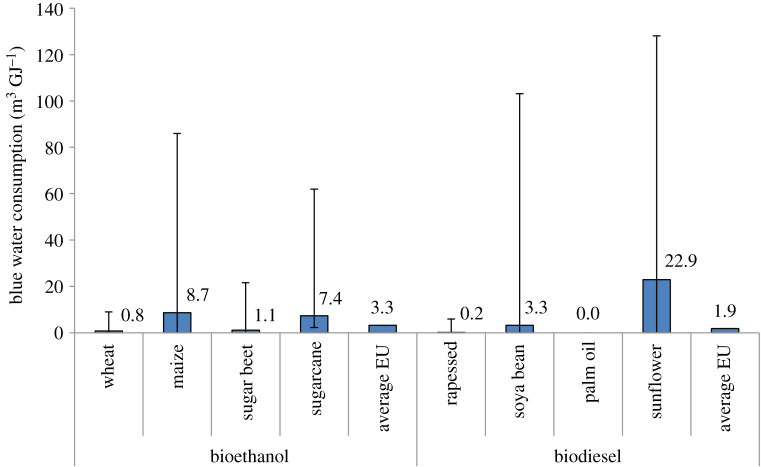
Blue water consumption for biofuels consumed in Europe. Based on data from [232]. Data labels represent the average values. (Online version in colour.)

A more recent study [232] that assessed the water footprint of first-generation biofuels consumed in Europe suggests that blue water consumption of biofuels is very diverse, depending on the underlying crop and country (figure 8). Bioethanol from sugar beet and wheat has lower water consumption because many countries produce crops using little or no irrigation. By contrast, the production of bioethanol from corn in Portugal consumes 86 m^3 ^GJ^−1^. Furthermore, while no irrigation is needed to cultivate crops for biodiesel in the UK, Poland and Germany, in Spain, on average 90 m^3^ of irrigation water is consumed to produce 1 GJ of crop-based biodiesel [232].

As indicated in figure 8, the average blue water consumption of bioethanol and biodiesel consumed in Europe is 3.3 m^3^ GJ^−1^ and 1.9 m^3^ GJ^−1^, respectively—this is 40 and 60 times higher compared to their respective fossil alternatives. If regional water stress is taken into account, as opposed to just the volume of water consumed, biofuels have water footprints a factor of 55–246 higher than fossil fuels [232]. This is a result of a large share of water consumption in the production of biofuels occurring in relatively water-stressed countries.

The blue water consumption of algae-based biofuels depends on the geographical location, production systems and conversion routes [233]. For example, the blue water consumption for biofuels produced in a closed photo-reactor in the Netherlands is estimated at 8 m^3 ^GJ^−1^, while it can be as high as 193 m^3^ GJ^−1^ if algae are cultivated in open pond systems in Hawaii [233]. There is also a difference between dry and wet conversion, with the blue water consumption being higher for the latter.

### (d) Biodiversity

Biofuels have the potential to contribute to loss of biodiversity through habitat loss and degradation, excessive nutrient load and other forms of pollution, over-exploitation and unsustainable use of land, as well as the cultivation of invasive alien species used as feedstocks [234]. The impact of biofuel production on biodiversity depends on the feedstock used and scale of production, management practices and LUC [235].

Intensive cultivation and use of agro-chemicals in the feedstock production for first-generation biofuels can create direct threats for local biodiversity [236,237]. LUC resulting from increased biofuel production exacerbates the risk of losing biodiversity through the direct loss of wildlife habitats, such as tropical rainforests [5,15].

Compared to first generation, second-generation biofuels are considered to have fewer negative impacts on biodiversity and could even have a positive effect [238]. For plant-based lignocellulosic feedstocks, this is because of their long growth cycle, low requirement for fertilizers and pesticides and less human intervention needed during the growth period. For example, large-scale SRC willow can provide benefits for some bird species, butterflies and flowering plants [239]. Furthermore, if degraded land is used for cultivation of feedstocks, the diversity of species might be enhanced. Similarly, perennial grasslands used for biomass production may enhance avian diversity, including migratory species. However, large energy crop monocultures can be detrimental to local biodiversity, particularly through habitat loss and the expansion of invasive species [5]. Eucalyptus, switchgrass and some *Miscanthus* species exhibit some traits of invasiveness [240].

The use of forest and agricultural residues as biofuel feedstock is expected to have a lower negative impact on biodiversity than dedicated energy crops [238]. Some of the impact on biodiversity associated with the use of forestry residues includes reduction in the amount of decaying wood—a niche habitat—and disturbance of wildlife caused by increased forest access. Excessive removal of agricultural residue from fields would also be a concern as it may increase weed growth, which could lead to the increased use of herbicides and thus affect local biodiversity.

For algal biofuels, the impact on biodiversity is uncertain. The large-scale cultivation of algae can bring significant risk to coastal biodiversity through invasion by algal species of coastal shallow ecosystems, such as mud flats, salt marshes, mangroves, sea grass bed and coral reefs [236].

Although the biodiversity loss is identified as one of the current key environmental concerns, it is only seldom included as an impact category in LCA studies of bioenergy systems [241]. Preserving biodiversity or avoiding biodiversity loss from biofuels is one of the criteria in sustainability certification schemes. However, biodiversity loss is difficult to measure and there are no standard ways of identifying and measuring systems that promote biodiversity.

### (e) Other environmental impacts

The LCA studies on biofuels have used different impact assessment methods to estimate the other environmental impacts. Therefore, it is difficult to compare them and provide a meaningful range of impacts for different biofuels. Furthermore, the studies differ in scope, with some considering the cradle-to-gate and others cradle-to-grave system boundary. Results of the latter studies also depend on assumptions regarding the type of vehicle in which biofuels are used. Nonetheless, several studies suggest that reduction in GHG emissions from biofuels compared to fossil fuels is carried out at the expense of other impacts, such as acidification and eutrophication [32,54,76,81,83,88,121,129,139,148,216,242–244].

These two impacts are compared in table 2 for different feedstocks relative to fossil fuels. As can be seen, first-generation bioethanol has up to three times higher acidification and 3–20 times higher eutrophication. Similarly, first-generation biodiesel has 30–70% higher acidification and 3–14 times greater eutrophication than the fossil alternative. These impacts are largely due to the use of fertilizers and associated emissions of acid gases and nutrients to air and water.

**Table 2.** Acidification and eutrophication of biofuels relative to fossil fuels.

biofuel type	feedstock	acidification^a^	eutrophication^a^	source
*first generation*				
bioethanol	corn	1.4–3	4.4–20	[242]
	wheat	3	5	[76]
	sugar beet	1.4–1.8	6–15	[83]
	sugarcane	2	2.8	[81]
biodiesel	rape seed	1.3–1.7	3.1–5	[32,54]
	soya bean	1.3–1.7	4–5	[121]
	palm oil	1.3	14	[88]
*second generation*				
bioethanol	short rotation coppice	0.45	1.2	[139]
	switchgrass	1.1	3.2	[148]
	straw	1.6–3	2–3.6	[129]
biodiesel	used cooking oil	0.2	0.63	[243]
	*Jatropha*	1	1	[244]
*third generation*				
biodiesel	algae	2.6–3	2.1–3.2	[216]

^a^The values represent the ratio of impacts from biofuels over fossil fuels and are dimensionless.

Lignocellulosic bioethanol from SCR performs better for acidification, but bioethanol from switchgrass and straw is worse than petrol for both impact categories. However, biodiesel from UCO has lower acidification and eutrophication than fossil diesel. These two impacts are also higher for algal biodiesel than for the fossil equivalent [216]. However, as mentioned earlier, the absence of full-scale plant data, large variability in production parameters and various assumptions lead to high uncertainty in the LCA estimates for algal biofuels [197].

## 4. Discussion

While many studies on biofuels have examined multiple scenarios and conducted sensitivity analyses, only a few have conducted comprehensive uncertainty analyses [63,197,245], demonstrating that the variability in results can be large. It is also clear from the findings discussed above that the outcomes of LCA studies are highly situational and dependent on many factors, including assumptions, data variation and gaps, models, methodology and software tools used. The outcomes of the study are also affected by the choice of allocation method, system boundaries and the cut-off criteria for auxiliary inputs. Especially in relation to GWP, there are significant uncertainties in models for estimating soil N_2_O emissions, direct and indirect LUC and the extent and duration of changes in soil and vegetation carbon stocks. The effects of these aspects on LCA results are discussed below.

### (a) Data

The problems related to data availability and quality are inherent to LCA. It is always preferable to use site-specific inventory data for developing LCA models of biofuels. However, data availability is often limited, particularly for second- and third-generation biofuels that, along with the associated process technologies, are still under development. As such, the use of unrepresentative data or assumptions to fill data gaps becomes a source of uncertainty [29]. There is also a great deal of technical, spatial and temporal variability associated with agronomic practices, such as fertilizer inputs, cultivation intensities and yields, as well as with biofuel conversion processes. LCA results are highly sensitive to variations in crop yields, use of nitrogen fertilizer and energy sources for biofuel conversion processes. Original and measured field data are still scarce and many studies rely on secondary data. There is also a room for improvement in existing LCA databases and a need to develop better, open access databases with common assumptions. Many data in common usage are reportedly out-of-date and finding new data is often difficult and time-consuming.

### (b) Methodological approaches

As mentioned earlier, ALCA and CLCA are different techniques that follow different methodologies and will normally have very different results that must be interpreted carefully based on the goal and scope of the study. For example, Searchinger *et al*. [246] found that using ALCA resulted in a 20% saving in GHG emissions from US corn ethanol compared to petrol. However, following a CLCA approach and considering the increase in output required by the US Energy Independence and Security Act lead to a 47% increase in emissions relative to petrol. This increase was related to LUC induced by higher prices of corn, soya bean and other grains as a consequence of the additional demand for corn for ethanol production.

As also mentioned earlier, CLCA is more suited for policy applications. However, the use of CLCA for policy is still in infancy and its application to biofuels is controversial and subject to criticism [29,247]. One of the main reasons is that consequential analysis is highly complex, being dependent on future projections, formulation of possible ‘what if’ scenarios and counterfactual circumstances, economic models of relationships between demand for inputs, price elasticities, supply and market effects of co-products, all of which can be highly uncertain [26,248,249]. There is also a real challenge in defining meaningful scenarios for how the world would develop with a biofuels policy or production in place. This is true for individual feedstocks all the way up to the economic and energy system models incorporated into CLCA studies. Therefore, caution should be exercised with the interpretation of CLCA results [249]. Furthermore, unlike ALCA, there is still no internationally agreed methodology for CLCA, making it difficult to carry out and compare different studies.

### (c) Allocation methods

Allocation is one of the most controversial issues in LCA. Both system expansion and allocation are subject to shortcomings: for system expansion, the difficulty is to estimate various substitution effects (similar to the related consequential issues in CLCA), while different allocation methods produce very different results. For instance, allocation by mass could result in the majority of impacts being allocated to the co-products rather than the biofuel which is the main (economic) product, while allocation by product cost/price leads to changes in the estimates of environmental impacts over time with variations in costs/prices without any other changes in the system. Several studies considered more than one allocation approach and found that the results were highly affected. For instance, some authors [17,132] showed that biofuels had significantly lower environmental impacts when using system expansion instead of allocation. In some cases, system expansion can lead to the negative values, suggesting net savings in environmental impacts, including in GHG emissions. However, studies assessing uncertainty in LCA of biofuels showed that system expansion also results in higher uncertainties [63,65]. Other authors found that environmental impacts were higher if economic allocation was used instead of mass and energy allocation [250]. For some biofuels, the co-products are sufficiently substantial that choice of allocation procedure can tip the balance between net benefit and net impact.

### (d) Emissions of soil nitrous oxide

Emissions of N_2_O arise from application of nitrogen fertilizer and decomposition of organic matter in soil. N_2_O is a potent GHG with a GWP 265 times higher than CO_2_ [41]; hence, its emission can have a significant effect on the GHG balance of biofuels. The N_2_O emissions are particularly significant for first-generation biofuel crops since fertilization rates are larger for these than for second-generation biofuels from perennial energy crops, which are usually grown without fertilizers, except during the initial establishment of the crop [251,252].

LCA studies often use the ‘Tier 1’ methodology developed by the Intergovernmental Panel for Climate Change (IPPC) to estimate N_2_O emissions from fertilizers [253]. According to this method, 1–1.5% of nitrogen in synthetic fertilizer applied to crops is emitted as N_2_O [253]. Since in reality, the occurrence and level of N_2_O emissions depend on many factors, including soil characteristics and local weather following fertilizer application on the soil, the default IPCC emission factors represent an uncertain estimate [23]. For example, a study by Crutzen *et al*. [254] suggested that N_2_O emissions in feedstock production can be three to five times higher than those estimated based on the IPCC methodology. Inclusion of these variable N_2_O rates leads to dramatically different estimates of GHG emissions in the life cycles of biofuels. For instance, for corn ethanol, the nitrogen conversion of 5% instead of 1.5% could change its GHG savings relative to petrol from around 40% to zero [255].

Conversely, a recent study in the UK concluded that N_2_O emissions averaged across arable land in the UK are below those determined by following the IPCC guidelines [256]. Compared to the default IPCC emissions factor of 1% (of the amount of nitrogen applied), direct N_2_O emissions from soil related to the use of fertilizers on crops for first-generation biofuels were estimated to be, on average, 0.46% of the nitrogen applied. However, the study noted that any one instance of fertilizer application is subject to interacting effects of rainfall and soil type, such that fertilizer-induced emissions could also be larger than the default IPCC emission factors in the wetter regions of the UK. Thus, in summary, the estimates of N_2_O emissions are highly variable and uncertain and should be treated with caution when interpreting the results.

### (e) Land-use change

An increasing global demand for biofuels highlighted the potential for the competition for land between cropland and natural ecosystems. Early LCA studies on biofuels, which excluded LUC, concluded that first-generation biofuels, such as corn ethanol, had lower GWP than petrol [257]. However, when attempts were made to account for the LUC effects of the expansion of first-generation biofuels, these findings came under question [43,246]. Since then, several other studies have cast doubt on the ability of first-generation biofuels to meet mandatory GHG savings targets if LUC is involved [119,258].

From an LCA perspective, DLUC is relatively straightforward and easy to include in the assessment, although the uncertainty remains high. However, estimating ILUC related to biofuels remains difficult, complex and highly uncertain [259,260]. The latter is exemplified by that fact that estimates of GHG emissions from ILUC range widely, from very small to very large [261]. For instance, a study on the ILUC associated with US corn ethanol found that the ILUC emissions varied from 10 to 340 g CO_2_ eq. MJ^−1^ [262]. For these reasons, the effects of ILUC and how to account for them in assessing the sustainability of biofuels are key areas requiring further research and consensus building [42,260]. Part of the challenge is constructing and analysing credible counterfactual scenarios. Another challenge is the economic (equilibrium) models used for consequential modelling [247,263] and the assumed yield-price elasticities for crops [26]. ILUC models make various assumption to estimate how much indirect change might be induced up to 20 years into the future under prescribed scenarios. Therefore, such estimates would only apply for the assumed conditions and must be interpreted with caution [264]. The lack of transparency in ILUC models, many of which are proprietary, is also problematic.

There is an ongoing question about how policymakers should respond to the growing evidence on ILUC from biofuel production. The blanket application of ‘ILUC factors’ according to feedstock type is unpopular as it offers producers no opportunity to improve the performance of their individual supply chains [265]. Moreover, there are many other drivers of LUC besides biofuels, such as demand for food and timber, urban development and infrastructure, leading some to argue that it is unfair to consider ILUC only for biofuels [247,266].

### (f) Soil organic carbon

SOC is one of the largest carbon pools in the terrestrial ecosystem [267]. Its balance is affected because of agricultural activities and LUC. Depending on various soil characteristics and agricultural practices, soil can act as either a sink or a source of carbon emissions. Soils may lose SOC by mineralization through cultivation, emitting CO_2_ to the atmosphere. Alternatively, SOC may increase through cropping or from repeated addition of crop residues or organic manures [268].

When biomass is left to decay in the soil, a part of the carbon in the biomass is sequestered into soil. Therefore, assuming biomass would have otherwise been left to decay in the soil, harvesting it decreases SOC and this may affect significantly the GHG balance of a biofuel [269,270]. For example, a study that included the effects of the removal of corn residue across the US corn belt concluded that the GWP of corn stover ethanol may exceed that of conventional petrol [271]. Another study on wheat-straw ethanol suggested that there is only a 30% probability that its GHG emissions will be 35% lower than that of petrol if SOC changes are included in the analysis [272]. A study on sugarcane ethanol claimed that the GHG balance of sugarcane ethanol could be significantly higher if the impacts on SOC from pre-harvest burning were considered [210]. The burning of biomass in the field, which is often carried out prior to a sugarcane harvest to help manual harvest, means that far less crop residues are left on the land to be incorporated into the soil.

Changes in SOC can also have a major influence on GHG emissions from LUC associated with biofuel feedstock production [267,273]. For example, reversal of grassland, woodland and perennial crops back to arable lands could reduce soil carbon by 0.6–1.7 t C ha^−1 ^yr^−1^, which would be emitted to the atmosphere as CO_2_ (2.2–6.2 t ha^−1^ yr^−1^). On the other hand, cultivation of perennial energy crops, such as SRC and *Miscanthus*, could sequester CO_2_ from the atmosphere into the soil at the rate of 2.2 t CO_2 _ha^−1^ yr^−1^ [267]. However, the sequestration potential is very site-specific and highly dependent on former and current agronomic practices, previous land use, as well as climate and soil characteristics [17,40,267,274–276]. Therefore, quantifying changes in SOC storage is an important factor in estimating GHG emissions of biofuels [277]. However, most LCA studies do not account for potential SOC changes from biomass cropping systems. This is probably due to inherent complexity of soil science, the high degree of intra- and inter-site variability, substantial data uncertainties and the challenges of linking biomass feedstock supply to specific soils [46]. Furthermore, there is no consensus in LCA on how to account for SOC change of agricultural activities and delayed GHG emissions [278]. However, the work on developing models to estimate SOC emissions related to biofuels is ongoing [273–275].

### (g) Biogenic carbon

In the context of biofuels, the term biogenic carbon refers to CO_2_ that is sequestered from the atmosphere during the growth of feedstocks and subsequently released during the combustion of the biofuel. ‘Carbon neutrality’ is achieved when CO_2_ sequestered and subsequently released are in balance. However, carbon neutrality cannot be claimed if there is a potential imbalance or a time delay between the amount of CO_2_ taken up during feedstock growth and the amount released through biofuel production and use. Since many bioenergy products—including annual crops and perennial grasses—have relatively short lifespans, carbon neutrality is commonly assumed in LCA standards and regulations. Hence, most LCA studies of biofuels assume that biogenic CO_2_ emissions, both from end-use combustion and the burning biomass to produce energy for conversion processes, are fully balanced by CO_2_ uptake during feedstock growth. While this assumption is reasonable for fuels from annual crops and perennial grass feedstocks, it is open to challenge in relation to biofuel production from feedstocks with harvest cycles of more than a few years—such as longer-lived lignocellulosic feedstocks from forestry [26,279]. For such feedstocks, it is important to consider the balance of carbon sequestered during feedstock growth versus that which is emitted during biofuel production and use, together with the overall time profile of biogenic carbon storage, emission and re-sequestration [279].

Different approaches to account for the temporal impact of carbon emissions are suggested in the literature; for example, carbon payback period, carbon discounting and time-integrated accounting of biogenic carbon [279,280]. Where accounting for the carbon storage in other, more long-lived bio-based products is required, there are various standards and methods [46] and these contain significant procedural differences. For example, GHG Protocol [281], PAS 2050 [282] and ISO 14067 [283] require reporting of emissions and removal of GHG emissions from biogenic carbon sources, while regulations the RED [8] and RFS [7] do not require such reporting. Furthermore, the time between the production of the product (storage of biogenic carbon) and its end of life (release of biogenic carbon), referred to as ‘delayed emissions’, varies among the standards. For instance, in PAS 2050 [282], all emissions that occur within a 100-year period are quantified and treated as if they occurred at the beginning of the time period. By contrast, ISO 14067 [283] makes a distinction between emissions released within and after the first 10 years.

### (h) Other environmental impacts

Production and use of biofuel generate emissions of various air pollutants, including particulate matter (PM), carbon monoxide (CO), nitrogen oxides (NO*x*), hydrocarbons and volatile organic compounds (VOCs). Unburned hydrocarbons, VOCs and NO*x* are precursors for the formation of summer smog and ground-level ozone. These pollutants are associated with increased morbidity and mortality from cardiovascular and respiratory diseases and certain cancers [284,285]. Air quality modelling studies show that life cycle emissions of some pollutants may be higher for biofuels when compared with fossil fuels, largely resulting from the emissions associated with feedstock production and biofuel processing [284,286]. For example, in the case of sugarcane ethanol in Brazil, burning of straw in fields is the common practice in certain areas and is the predominant source of PM [284,286]. Studies on health impacts of sugarcane ethanol in Brazil suggest that there is strong evidence that burning straw in sugarcane fields causes substantial respiratory diseases, such as asthma and pneumonia, in sugarcane fieldworkers and local populations [284,286–289]. These effects are often ignored in LCA studies.

In cradle-to-grave LCA studies, assessing impacts of vehicular exhaust emissions is another challenge as they are affected by many different parameters, including the type of engine and how it is run (the operational drive cycle), vehicle age and maintenance, the quality of the base fuel and exhaust after treatment [290]. Vehicular exhaust emissions of bioethanol blends vary with blend strength. However, in general, lower bioethanol blends (E5–E15) have lower CO and PM emissions compared to petrol [290,291]. Beer *et al*. [291] suggest that lower PM emissions from low-ethanol blends used in spark-ignition vehicles have slight health benefits over petrol. However, they lead to significantly higher emissions of acetaldehyde, which is one of the precursor VOCs involved in ground-level ozone formation. Similarly, higher ethanol blends (E85) lead to comparable, or slightly lower, levels of PM, NO*x* and CO emissions than petrol, but 5–10 times higher acetaldehyde emissions [290,292,293].

Compared to fossil diesel, biodiesel has generally lower exhaust emissions of PM, CO, hydrocarbons and VOCs, but higher NO*x* emissions [294,295]. These differences are small for 5–20% biodiesel blends and would lead to negligible or non-measurable impacts on air quality [294], but increase with higher blends [290]. On the other hand, Larcombe *et al*. [296] argue that, despite having lower PM emissions, biodiesel exhaust emissions could potentially be more harmful to human health because of higher proportion of ultra-fine particles (less than 100 nm diameter) compared to diesel exhaust. This is due to the fact that smaller particles remain suspended in the air for longer, are more easily inhaled and are able to penetrate more deeply into the lungs. However, other assessments on the potential human health implications of biodiesel suggest that the use of biodiesel fuel blends compared to fossil diesel results in minimal changes in health impacts [294,295]. Thus, the topic of human health impacts from biofuels remains open to debate, requiring further research and evidence.

Besides air pollution, production of liquid biofuels could affect human health directly through water and soil pollution and occupational hazards [284]. However, these effects are scarcely discussed in the literature and should be explored further to understand whether there are risks that need to be addressed.

## 5. Conclusion and recommendations

LCA is widely used as a tool to estimate GWP and other environmental impacts of biofuels. However, as evident from this review, the estimates vary widely among the studies owing to a wide range of methodological choices in LCA and various uncertainties. Despite this, the existing evidence base is instructive. Firstly, it shows that, if no LUC is involved, first-generation biofuels can—on average—have lower GHG emissions than fossil fuels, but GHG savings for most of the feedstocks are not sufficient to meet those required by the RED. Secondly, in general, second-generation biofuels have a greater potential than first generation to reduce GHG emissions, again provided there is no LUC. However, the development of second-generation biofuels will take time and is likely to depend on the continued support of first-generation fuels to give the industry the confidence to invest. Thirdly, it is also clear that, at present state of development, third-generation biofuels from algae are unlikely to make a contribution to the transport sector as their GHG emissions are higher than those from fossil fuels. Moreover, they are unproven and expensive to produce and, as such, the algal feedstock will continue to be restricted to high-value markets, such as cosmetics and dietary supplements.

LCA is a complex tool that lies at the interface between science, engineering and policy. Despite this complexity, it is often perceived as a tool that can give a definitive answer to multifaceted questions. As the findings in this review demonstrate clearly, there are no definitive answers. Even focusing only on the GWP of biofuels—one of the main drivers for their development—brings with it a host of uncertainties. Moreover, almost every aspect related to biofuels is dynamic in nature across different scales, which adds to the complexity. Examples include changes in soil carbon content over time (micro-scale); time needed to replace vegetation used as feedstock for biofuels (meso-scale); and development of global biofuel supply chains (macro-scale). Considering these dynamic aspects and their interconnections presents a considerable challenge. There are also significant uncertainties in the models for estimating direct and indirect LUC, changes in SOC stocks and N_2_O emissions. It is important to recognize these limitations and interpret the results accordingly.

In addition to the environmental impacts, there are many other sustainability issues that must be considered when assessing the sustainability of biofuels. These include: costs of production and competitiveness with fossil fuels; food, energy and water security; employment provision; rural development; and human health impacts. It is essential that the sustainability aspects of biofuels be evaluated on a life cycle basis across full supply chains to avoid shifting the burdens from one part of the life cycle or supply chain to another. It is also important to note that LCA and wider sustainability assessments are of little use if the results cannot be trusted. Therefore, strong auditing of biofuel supply chains is vital to prevent negative socio-economic effects as well as to ensure traceability of the fuels and to mitigate the risk of fraud. Moreover, improving transparency, data availability and sharing are key if LCA is to be trusted and useful for policy. This could be achieved through development of open national and global databases, in a similar way that national inventories have been developed for GHG reporting under the Kyoto Protocol. It is also important to ensure that the data and models from different disciplines that are used in LCA preserve reasonable levels of transparency, rigour and robustness to avoid misuse and misinterpretation.

ALCA studies, which account for the direct impacts, should follow the ISO 14040 and 14044 standards more rigorously. For CLCA, both methodological and practical aspects need improvements. For the former, further work is required towards the standardization of CLCA methodology. As part of that, there is a need to improve development of counterfactual (what if) scenarios and ILUC models. Involvement of multiple stakeholders can help to build consensus on the definition of the scenarios and to improve the transparency of ILUC models, their assumptions and the associated uncertainty. In addition to improving the CLCA methodology, much work is required in its application in practice. Specifically, there is a need to validate ILUC models with empirical evidence; empirical methods to test alternative hypotheses also require attention. Further work is also needed on the development of models and empirical evidence of changes in soil and plant carbon stocks as well as emissions of nitrous oxide related to the application of fertilizers. Research is also needed on estimations of biogenic carbon, particularly changes in the forest carbon stock that may be affected by an increase in biofuels demand.

It is also important to take into account that biofuels do not exist in isolation but are part of much wider systems, including energy, agriculture and forestry. Like other production systems with which they interact, biofuels impact on various ecosystem services, such as land, water and food. It is, therefore, essential to take an integrated, systems view to developing future policy to ensure that biofuels are not disadvantaged relative to other sectors or that progress made in this sector is not undone by unsustainable practices in others. Analysis and, ultimately, policies based on ecosystem services and natural capital at a landscape level are needed to make the best overall use of land. This would, in turn, optimize ecosystem services, such as carbon storage, biodiversity, reductions of agricultural run-off and increases in water quality and flood risk management. Complete value chains rather than single bioenergy products should be analysed together to understand the interactions across sectors and land uses with the goal of identifying opportunities where collective benefits can be realized.

## Supplementary Material

Detailed data on GHG emissions from different biofuels

## Data accessibility

This article does not contain any additional data.

## References

[1] Sims R *et al* 2014 Transport. In Climate change 2014: mitigation of climate change contribution of working group III to the fifth assessment report of the intergovernmental panel on climate change (eds Edenhofer O et al.). Cambridge, UK and New York, NY: Cambridge University Press.

[2] IEA. 2019 Renewables 2019. Paris See https://www.iea.org/reports/renewables-2019.

[3] Michael K, Steffi N, Peter D 2011 The past, present, and future of biofuels—biobutanol as promising alternative. In Biofuel production—recent developments and prospects (ed. dos Santos MA), pp. 451–486. Rijeka, Croatia: InTech.

[4] Soccol CR, Vandenberghe LPS, Costa B, Woiciechowski AL, de Carvalho JC, Medeiros ABP, Francisco AM, Bonomi LJ 2005 Brazilian biofuel program: an overview. J. Sci. Ind. Res. 64, 897–904.

[5] Food and Agriculture Organization (FAO). 2013 Biofuels and the sustainability challenge: a global assessment of sustainability issues, trends and policies for biofuels and related feedstocks. Rome, Italy: Food and Agriculture Organization of the United Nations.

[6] International Renewable Energy Agency (IRENA). 2016 Innovation outlook: advanced liquid biofuels. Abu Dhabi, United Arab Emirates: International Renewable Energy Agency.

[7] EPA. 2010 Renewable Fuel Standard Program (RFS2) Regulatory Impact Analysis (EPA-420-R-10-006). U.S. Environmental Protection Agency, Office of Transportation and Air Quality, Assessment and Standards Division See www.epa.gov/otaq/fuels/renewablefuels/regulations.htm

[8] European Commission. 2018 Directive (EU) 2018/2001 of the European Parliament and of the Council of 11 December 2018 on the promotion of the use of energy from renewable sources. Brussels, Belgium: Official Journal of the European Union.

[9] Renewable Energy Policy Network for the 21st Century (REN21). 2019 Renewables 2019—Global status report See https://www.ren21.net/wp-content/uploads/2019/05/gsr_2019_full_report_en.pdf.

[10] OECD/FAO. 2019 OECD-FAO agricultural outlook 2019–2028. Paris, France: OECD Publishing.

[11] IEA. 2011 Technology roadmap: biofuels for transport. Paris, France: International Energy Agency.

[12] Timilsina GR 2014 Biofuels in the long-run global energy supply mix for transportation. Phil. Trans. R. Soc. A 372, 20120323 (10.1098/rsta.2012.0323) 24298077

[13] Searle S, Malins C 2015 A reassessment of global bioenergy potential in 2050. GCB Bioenergy 7, 328–336. (10.1111/gcbb.12141)

[14] Azapagic A, Stichnothe H 2011 Assessing sustainability of biofuels. In Sustainable development in practice: case studies for engineers and scientists, 2nd edn (eds Azapagic A, Perdan S). Chichester, UK: John Wiley & Sons.

[15] UNEP. 2009 Towards sustainable production and use of resources: assessing biofuels. United Nations Environment Programme See https://www.resourcepanel.org/file/560/download?token=04PkF6fe.

[16] Roy P, Dutta A 2013 Life cycle assessment of ethanol derived from sawdust. Bioresour. Technol. 150, 407–411. (10.1016/j.biortech.2013.08.057) 23993286

[17] Stephenson AL, Dupree P, Scott SA, Dennis JS 2010 The environmental and economic sustainability of potential bioethanol from willow in the UK. Bioresour. Technol. 101, 9612–9623. (10.1016/j.biortech.2010.07.104) 20727740

[18] Daystar J, Treasure T, Gonzalez R, Reeb C, Venditti R, Kelley S 2015 The NREL biochemical and thermochemical ethanol conversion processes: financial and environmental analysis comparison. Bioresources 10, 5096–5116. (10.15376/biores.10.3.5096-5116)

[19] Passell H et al 2013 Algae biodiesel life cycle assessment using current commercial data. J. Environ. Manage. 129, 103–111. (10.1016/j.jenvman.2013.06.055) 23900083

[20] Shonnard DR, Klemetsrud B, Sacramento-Rivero J, Navarro-Pineda F, Hilbert J, Handler R, Suppen N, Donovan RP 2015 A review of environmental life cycle assessments of liquid transportation biofuels in the Pan American region. Environ. Manage. 56, 1356–1376. (10.1007/s00267-015-0543-8) 26041501

[21] Morales M, Quintero J, Conejeros R, Aroca G 2015 Life cycle assessment of lignocellulosic bioethanol: environmental impacts and energy balance. Renewable Sustainable Energy Rev. 42, 1349–1361. (10.1016/j.rser.2014.10.097)

[22] Roy P, Tokuyasu K, Orikasa T, Nakamura N, Shiina T 2012 A review of life cycle assessment (LCA) of bioethanol from lignocellulosic biomass. Jarq-Jpn Agric. Res. Q. 46, 41–57. (10.6090/jarq.46.41)

[23] Menten F, Cheze B, Patouillard L, Bouvart F 2013 A review of LCA greenhouse gas emissions results for advanced biofuels: the use of meta-regression analysis. Renew. Sustain. Energy Rev. 26, 108–134. (10.1016/j.rser.2013.04.021)

[24] Sieverding HL, Bailey LM, Hengen TJ, Clay DE, Stone JJ 2015 Meta-analysis of soybean-based biodiesel. J. Environ. Qual. 44, 1038–1048. (10.2134/jeq2014.07.0320) 26437085

[25] van Eijck J, Romijn H, Balkema A, Faaij A 2014 Global experience with jatropha cultivation for bioenergy: an assessment of socio-economic and environmental aspects. Renew. Sustain. Energy Rev. 32, 869–889. (10.1016/j.rser.2014.01.028)

[26] Sanchez ST, Woods J, Akhurst M, Brander M, O'Hare M, Dawson TP, Edwards R, Liska AJ, Malpas R 2012 Accounting for indirect land-use change in the life cycle assessment of biofuel supply chains. J. R. Soc. Interface. 9, 1105–1119. (10.1098/rsif.2011.0769) 22467143 PMC3350742

[27] Plevin RJ, Delucchi MA, Creutzig F 2014 Using attributional life cycle assessment to estimate climate-change mitigation benefits misleads policy makers. J. Ind. Ecol. 18, 73–83. (10.1111/jiec.12074)

[28] ISO. 2006 ISO 14040:2006 environmental management—life cycle assessment—principles and framework. London, UK: BSI.

[29] McManus MC, Taylor CM, Mohr A, Whittaker C, Scown CD, Borrion AL, Glithero NJ, Yin Y 2015 Challenge clusters facing LCA in environmental decision-making—what we can learn from biofuels. Int. J. Life Cycle Assess. 20, 1399–1414. (10.1007/s11367-015-0930-7) 27453635 PMC4939404

[30] Singh A, Pant D, Korres NE, Nizami A-S, Prasad S, Murphy JD 2010 Key issues in life cycle assessment of ethanol production from lignocellulosic biomass: challenges and perspectives. Bioresour. Technol. 101, 5003–5012. (10.1016/j.biortech.2009.11.062) 20015644

[31] Wiloso EI, Heijungs R, de Snoo GR 2012 LCA of second generation bioethanol: a review and some issues to be resolved for good LCA practice. Renew. Sustain. Energy Rev. 16, 5295–5308. (10.1016/j.rser.2012.04.035)

[32] Iriarte A, Rieradevall J, Gabarrell X 2012 Transition towards a more environmentally sustainable biodiesel in South America: the case of Chile. Appl. Energy. 91, 263–273. (10.1016/j.apenergy.2011.09.024)

[33] Sreekumar A, Shastri Y, Wadekar P, Patil M, Lali A 2020 Life cycle assessment of ethanol production in a rice-straw-based biorefinery in India. Clean Technol. Environ. Policy 22, 409–422. (10.1007/s10098-019-01791-0)

[34] Mandade P, Bakshi BR, Yadav GD 2015 Ethanol from Indian agro-industrial lignocellulosic biomass—a life cycle evaluation of energy, greenhouse gases, land and water. Int. J. Life Cycle Assess. 20, 1649–1658. (10.1007/s11367-015-0966-8)

[35] Kesieme U, Pazouki K, Murphy A, Chrysanthou A 2019 Attributional life cycle assessment of biofuels for shipping: addressing alternative geographical locations and cultivation systems. J. Environ. Manage. 235, 96–104. (10.1016/j.jenvman.2019.01.036) 30677660

[36] Prasara-A J, Grant T 2011 Comparative life cycle assessment of uses of rice husk for energy purposes. Int. J. Life Cycle Assess. 16, 493–502. (10.1007/s11367-011-0293-7)

[37] Moghaddam EA, Ahlgren S, Hulteberg C, Nordberg A 2015 Energy balance and global warming potential of biogas-based fuels from a life cycle perspective. Fuel Process. Technol. 132, 74–82. (10.1016/j.fuproc.2014.12.014)

[38] Garba NA, Duckers LJ, Hall WJ 2014 Climate change impacts on life cycle greenhouse gas (GHG) emissions savings of biomethanol from corn and soybean. Int. J. Life Cycle Assess. 19, 806–813. (10.1007/s11367-013-0680-3)

[39] Lim S, Lee KT 2011 Parallel production of biodiesel and bioethanol in palm-oil-based biorefineries: life cycle assessment on the energy and greenhouse gases emissions. Biofuels Bioprod. Biorefin.-Biofpr. 5, 132–150. (10.1002/bbb.271)

[40] Cherubini F, Jungmeier G 2010 LCA of a biorefinery concept producing bioethanol, bioenergy, and chemicals from switchgrass. Int. J. Life Cycle Assess. 15, 53–66. (10.1007/s11367-009-0124-2)

[41] IPCC. 2013 Climate change 2013: the physical science basis. Contribution of working group I to the fifth assessment report of the intergovernmental panel on climate change. Cambridge, UK: Intergovernmental Panel on Climate Change.

[42] Harris ZM, Spake R, Taylor G 2015 Land use change to bioenergy: a meta-analysis of soil carbon and GHG emissions. Biomass Bioenergy 82, 27–39. (10.1016/j.biombioe.2015.05.008)

[43] Fargione J, Hill J, Tilman D, Polasky S, Hawthorne P 2008 Land clearing and the biofuel carbon debt. Science 319, 1235–1238. (10.1126/science.1152747) 18258862

[44] Mello FFC et al 2014 Payback time for soil carbon and sugar-cane ethanol. Nat. Clim. Change 4, 605–609. (10.1038/nclimate2239)

[45] Borjesson P, Tufvesson LM 2011 Agricultural crop-based biofuels—resource efficiency and environmental performance including direct land use changes. J. Clean. Prod. 19, 108–120. (10.1016/j.jclepro.2010.01.001)

[46] Pawelzik P et al 2013 Critical aspects in the life cycle assessment (LCA) of bio-based materials—reviewing methodologies and deriving recommendations. Resour. Conserv. Recycling. 73, 211–228. (10.1016/j.resconrec.2013.02.006)

[47] Ou X, Zhang X, Chang S, Guo Q 2009 Energy consumption and GHG emissions of six biofuel pathways by LCA in (the) People's Republic of China. Appl. Energy. 86, S197–S208. (10.1016/j.apenergy.2009.04.045)

[48] Stephenson AL, von Blottnitz H, Brent AC, Dennis JS, Scott SA 2010 Global warming potential and fossil-energy requirements of biodiesel production scenarios in South Africa. Energy Fuels 24, 2489–2499. (10.1021/ef100051g)

[49] Tomaschek J, Oezdemir ED, Fahl U, Eltrop L 2012 Greenhouse gas emissions and abatement costs of biofuel production in South Africa. Global Change Biol. Bioenergy 4, 799–810. (10.1111/j.1757-1707.2011.01154.x)

[50] Knoope MMJ, Balzer CH, Worrell E 2019 Analysing the water and greenhouse gas effects of soya bean-based biodiesel in five different regions. GCB Bioenergy 11, 381–399. (10.1111/gcbb.12558)

[51] Harsono SS, Prochnow A, Grundmann P, Hansen A, Hallmann C 2012 Energy balances and greenhouse gas emissions of palm oil biodiesel in Indonesia. GCB Bioenergy 4, 213–228. (10.1111/j.1757-1707.2011.01118.x)

[52] Prapaspongsa T, Musikavong C, Gheewala SH 2017 Life cycle assessment of palm biodiesel production in Thailand: impacts from modelling choices, co-product utilisation, improvement technologies, and land use change. J. Clean. Prod. 153, 435–447. (10.1016/j.jclepro.2017.03.130)

[53] Gallejones P, Pardo G, Aizpurua A, del Prado A 2015 Life cycle assessment of first-generation biofuels using a nitrogen crop model. Sci. Total Environ. 505, 1191–1201. (10.1016/j.scitotenv.2014.10.061) 25461117

[54] Gonzalez-Garcia S, Garcia-Rey D, Hospido A 2013 Environmental life cycle assessment for rapeseed-derived biodiesel. Int. J. Life Cycle Assess. 18, 61–76. (10.1007/s11367-012-0444-5)

[55] Iriarte A, Villalobos P 2013 Greenhouse gas emissions and energy balance of sunflower biodiesel: identification of its key factors in the supply chain. Resour. Conserv. Recycling 73, 46–52. (10.1016/j.resconrec.2013.01.014)

[56] Castanheira ÉG, Freire F 2017 Environmental life cycle assessment of biodiesel produced with palm oil from Colombia. Int. J. Life Cycle Assess. 22, 587–600. (10.1007/s11367-016-1097-6)

[57] Martinez-Hernandez E, Ibrahim MH, Leach M, Sinclair P, Campbell GM, Sadhukhan J 2013 Environmental sustainability analysis of UK whole-wheat bioethanol and CHP systems. Biomass Bioenergy 50, 52–64. (10.1016/j.biombioe.2013.01.001)

[58] García CA, Fuentes A, Hennecke A, Riegelhaupt E, Manzini F, Masera O 2011 Life-cycle greenhouse gas emissions and energy balances of sugarcane ethanol production in Mexico. Appl. Energy. 88, 2088–2097. (10.1016/j.apenergy.2010.12.072)

[59] Soam S, Kumar R, Gupta RP, Sharma PK, Tuli DK, Das B 2015 Life cycle assessment of fuel ethanol from sugarcane molasses in northern and western India and its impact on Indian biofuel programme. Energy 83, 307–315. (10.1016/j.energy.2015.02.025)

[60] Tsiropoulos I et al 2014 Life cycle assessment of sugarcane ethanol production in India in comparison to Brazil. Int. J. Life Cycle Assess. 19, 1049–1067. (10.1007/s11367-014-0714-5)

[61] Weinberg J, Kaltschmitt M 2013 Greenhouse gas emissions from first generation ethanol derived from wheat and sugar beet in Germany—analysis and comparison of advanced by-product utilization pathways. Appl. Energy. 102, 131–139. (10.1016/j.apenergy.2012.06.047)

[62] Styles D, Gibbons J, Williams AP, Dauber J, Stichnothe H, Urban B, Chadwick DR, Jones DL 2015 Consequential life cycle assessment of biogas, biofuel and biomass energy options within an arable crop rotation. Global Change Biol. Bioenergy 7, 1305–1320. (10.1111/gcbb.12246)

[63] Yan X, Boies AM 2013 Quantifying the uncertainties in life cycle greenhouse gas emissions for UK wheat ethanol. Environ. Res. Lett. 8, 015024 (10.1088/1748-9326/8/1/015024)

[64] Hassan MNA, Jaramillo P, Griffin WM 2011 Life cycle GHG emissions from Malaysian oil palm bioenergy development: the impact on transportation sector's energy security. Energy Policy 39, 2615–2625. (10.1016/j.enpol.2011.02.030)

[65] Kim S, Dale BE 2009 Regional variations in greenhouse gas emissions of biobased products in the United States—corn-based ethanol and soybean oil. Int. J. Life Cycle Assess. 14, 540–546. (10.1007/s11367-009-0106-4)

[66] Muñoz I, Flury K, Jungbluth N, Rigarlsford G, Canals LM, King H 2014 Life cycle assessment of bio-based ethanol produced from different agricultural feedstocks. Int. J. Life Cycle Assess. 19, 109–119. (10.1007/s11367-013-0613-1)

[67] Walter A, Dolzan P, Quilodrán O, de Oliveira JG, da Silva C, Piacente F, Segerstedt A 2011 Sustainability assessment of bio-ethanol production in Brazil considering land use change, GHG emissions and socio-economic aspects. Energy Policy 39, 5703–5716. (10.1016/j.enpol.2010.07.043)

[68] Wang M, Han J, Dunn JB, Cai H, Elgowainy A 2012 Well-to-wheels energy use and greenhouse gas emissions of ethanol from corn, sugarcane and cellulosic biomass for US use. Environ. Res. Lett. 7, 045905 (10.1088/1748-9326/7/4/045905)

[69] Reinhard J, Zah R 2009 Global environmental consequences of increased biodiesel consumption in Switzerland: consequential life cycle assessment. J. Clean. Prod. 17(Suppl. 1), S46–S56. (10.1016/j.jclepro.2009.05.003)

[70] Meijide A et al 2020 Measured greenhouse gas budgets challenge emission savings from palm-oil biodiesel. Nat. Commun. 11, 1089 (10.1038/s41467-020-14852-6) 32107373 PMC7046764

[71] Prapaspongsa T, Gheewala SH 2017 Consequential and attributional environmental assessment of biofuels: implications of modelling choices on climate change mitigation strategies. Int. J. Life Cycle Assess. 22, 1644–1657. (10.1007/s11367-017-1355-2)

[72] Reijnders L, Huijbregts MAJ 2008 Biogenic greenhouse gas emissions linked to the life cycles of biodiesel derived from European rapeseed and Brazilian soybeans. J. Clean. Prod. 16, 1943–1948. (10.1016/j.jclepro.2008.01.012)

[73] Canter CE, Dunn JB, Han J, Wang Z, Wang M 2016 Policy implications of allocation methods in the life cycle analysis of integrated corn and corn stover ethanol production. Bioenergy Res. 9, 77–87. (10.1007/s12155-015-9664-4)

[74] Liska AJ, Yang HS, Bremer VR, Klopfenstein TJ, Walters DT, Erickson GE, Cassman KG 2009 Improvements in life cycle energy efficiency and greenhouse gas emissions of corn-ethanol. J. Ind. Ecol. 13, 58–74. (10.1111/j.1530-9290.2008.00105.x)

[75] Kauffman N, Hayes D, Brown R 2011 A life cycle assessment of advanced biofuel production from a hectare of corn. Fuel 90, 3306–3314. (10.1016/j.fuel.2011.06.031)

[76] Belboom S, Bodson B, Léonard A 2015 Does the production of Belgian bioethanol fit with European requirements on GHG emissions? Case of wheat. Biomass Bioenergy 74, 58–65. (10.1016/j.biombioe.2015.01.005)

[77] Buchspies B, Kaltschmitt M 2016 Life cycle assessment of bioethanol from wheat and sugar beet discussing environmental impacts of multiple concepts of co-product processing in the context of the European Renewable Energy Directive. Biofuels 7, 141–153. (10.1080/17597269.2015.1122472)

[78] Elsgaard L, Olesen JE, Hermansen JE, Kristensen IT, Borgesen CD 2013 Regional greenhouse gas emissions from cultivation of winter wheat and winter rapeseed for biofuels in Denmark. Acta Agric. Scand. B 63, 219–230. (10.1080/09064710.2012.751451)

[79] Wang L, Quiceno R, Price C, Malpas R, Woods J 2014 Economic and GHG emissions analyses for sugarcane ethanol in Brazil: looking forward. Renewable Sustainable Energy Rev. 40, 571–582. (10.1016/j.rser.2014.07.212)

[80] Souza SP, Seabra JEA 2014 Integrated production of sugarcane ethanol and soybean biodiesel: environmental and economic implications of fossil diesel displacement. Energy Convers. Manage. 87, 1170–1179. (10.1016/j.enconman.2014.06.015)

[81] Cavalett O, Chagas MF, Seabra JEA, Bonomi A 2013 Comparative LCA of ethanol versus gasoline in Brazil using different LCIA methods. Int. J. Life Cycle Assess. 18, 647–658. (10.1007/s11367-012-0465-0)

[82] Seabra JEA, Macedo IC, Chum HL, Faroni CE, Sarto CA 2011 Life cycle assessment of Brazilian sugarcane products: GHG emissions and energy use. Biofuels Bioprod. Biorefin 5, 519–532. (10.1002/bbb.289)

[83] Bessou C, Lehuger S, Gabrielle B, Mary B 2013 Using a crop model to account for the effects of local factors on the LCA of sugar beet ethanol in Picardy region, France. Int. J. Life Cycle Assess. 18, 24–36. (10.1007/s11367-012-0457-0)

[84] Halleux H, Lassaux S, Renzoni R, Germain A 2008 Comparative life cycle assessment of two biofuels ethanol from sugar beet and rapeseed methyl ester. Int. J. Life Cycle Assess. 13, 184–190. (10.1065/lca2008.03.382)

[85] Souza SP, Gopal AR, Seabra JEA 2015 Life cycle assessment of biofuels from an integrated Brazilian algae-sugarcane biorefinery. Energy 81, 373–381. (10.1016/j.energy.2014.12.050)

[86] Abdul-Manan AFN 2017 Lifecycle GHG emissions of palm biodiesel: Unintended market effects negate direct benefits of the Malaysian Economic Transformation Plan (ETP). Energy Policy 104, 56–65. (10.1016/j.enpol.2017.01.041)

[87] Malca J, Coelho A, Freire F 2014 Environmental life-cycle assessment of rapeseed-based biodiesel: alternative cultivation systems and locations. Appl. Energy. 114, 837–844. (10.1016/j.apenergy.2013.06.048)

[88] Arpornpong N, Sabatini DA, Khaodhiar S, Charoensaeng A 2015 Life cycle assessment of palm oil microemulsion-based biofuel. Int. J. Life Cycle Assess. 20, 913–926. (10.1007/s11367-015-0888-5)

[89] Arvidsson R, Persson S, Froling M, Svanstrom M 2011 Life cycle assessment of hydrotreated vegetable oil from rape, oil palm and Jatropha. J. Clean. Prod. 19, 129–137. (10.1016/j.jclepro.2010.02.008)

[90] Intarapong P, Papong S, Malakul P 2016 Comparative life cycle assessment of diesel production from crude palm oil and waste cooking oil via pyrolysis. Int. J. Energy Res. 40, 702–713. (10.1002/er.3433)

[91] Pehnelt G, Vietze C 2013 Recalculating GHG emissions saving of palm oil biodiesel. Environ. Dev. Sustain. 15, 429–479. (10.1007/s10668-012-9387-z)

[92] de Souza SP, Pacca S, de Ávila MT, Borges JLB 2010 Greenhouse gas emissions and energy balance of palm oil biofuel. Renew. Energy 35, 2552–2561. (10.1016/j.renene.2010.03.028)

[93] Pleanjai S, Gheewala SH 2009 Full chain energy analysis of biodiesel production from palm oil in Thailand. Appl. Energy 86(Suppl. 1), S209–S214. (10.1016/j.apenergy.2009.05.013)

[94] Ukaew S, Beck E, Archer DW, Shonnard DR 2015 Estimation of soil carbon change from rotation cropping of rapeseed with wheat in the hydrotreated renewable jet life cycle. Int. J. Life Cycle Assess. 20, 608–622. (10.1007/s11367-015-0863-1)

[95] Garrain D, Herrera I, Lechon Y, Lago C 2014 Well-to-tank environmental analysis of a renewable diesel fuel from vegetable oil through co-processing in a hydrotreatment unit. Biomass Bioenergy 63, 239–249. (10.1016/j.biombioe.2014.01.035)

[96] Kalnes TN, Koers KP, Marker T, Shonnard DR 2009 A technoeconomic and environmental life cycle comparison of green diesel to biodiesel and syndiesel. Environ. Prog. Sustain. Energy 28, 111–120. (10.1002/ep.10319)

[97] Hou J, Zhang P, Yuan X, Zheng Y 2011 Life cycle assessment of biodiesel from soybean, jatropha and microalgae in China conditions. Renew. Sustain. Energy Rev. 15, 5081–5091. (10.1016/j.rser.2011.07.048)

[98] Spinelli D, Jez S, Pogni R, Basosi R 2013 Environmental and life cycle analysis of a biodiesel production line from sunflower in the Province of Siena (Italy). Energy Policy 59, 492–506. (10.1016/j.enpol.2013.04.009)

[99] Yee KF, Tan KT, Abdullah AZ, Lee KT 2009 Life cycle assessment of palm biodiesel: revealing facts and benefits for sustainability. Appl. Energy. 86, S189–S196. (10.1016/j.apenergy.2009.04.014)

[100] Herrmann IT, Jørgensen A, Bruun S, Hauschild MZ 2013 Potential for optimized production and use of rapeseed biodiesel. Based on a comprehensive real-time LCA case study in Denmark with multiple pathways. Int. J. Life Cycle Assess. 18, 418–430. (10.1007/s11367-012-0486-8)

[101] Acquaye AA et al 2011 Identification of ‘carbon hot-spots’ and quantification of GHG intensities in the biodiesel supply chain using hybrid LCA and structural path analysis. Environ. Sci. Technol. 45, 2471–2478. (10.1021/es103410q) 21319814

[102] Buchspies B, Kaltschmitt M 2018 A consequential assessment of changes in greenhouse gas emissions due to the introduction of wheat straw ethanol in the context of European legislation. Appl. Energy. 211, 368–381. (10.1016/j.apenergy.2017.10.105)

[103] Klein BC, Chagas MF, Watanabe MDB, Bonomi A, Maciel FR 2019 Low carbon biofuels and the New Brazilian National Biofuel Policy (RenovaBio): a case study for sugarcane mills and integrated sugarcane-microalgae biorefineries. Renew. Sustain. Energy Rev. 115, 109 365 (10.1016/j.rser.2019.109365)

[104] Maga D, Thonemann N, Hiebel M, Sebastião D, Lopes TF, Fonseca C, Girio F 2019 Comparative life cycle assessment of first- and second-generation ethanol from sugarcane in Brazil. Int. J. Life Cycle Assess. 24, 266–280. (10.1007/s11367-018-1505-1)

[105] Vera I, Hoefnagels R, van der Kooij A, Moretti C, Junginger M 2020 A carbon footprint assessment of multi-output biorefineries with international biomass supply: a case study for the Netherlands. Biofuels Bioprod. Biorefin. 14, 198–224. (10.1002/bbb.2052)

[106] Alexiades A, Kendall A, Winans KS, Kaffka SR 2018 Sugar beet ethanol (*Beta vulgaris* L.): a promising low-carbon pathway for ethanol production in California. J. Clean. Prod. 172, 3907–3917. (10.1016/j.jclepro.2017.05.059)

[107] Ratthanaphra D, Suwanmanee U 2019 Uncertainty analysis of environmental sustainability of biodiesel production using Thai domestic rare earth oxide solid catalysts. Sustain. Prod. Consump. 18, 237–249. (10.1016/j.spc.2019.01.001)

[108] Chen R, Qin Z, Han J, Wang M, Taheripour F, Tyner W, O'Connor D, Duffield J 2018 Life cycle energy and greenhouse gas emission effects of biodiesel in the United States with induced land use change impacts. Bioresour. Technol. 251, 249–258. (10.1016/j.biortech.2017.12.031) 29287277

[109] O'Keeffe S, Majer S, Drache C, Franko U, Thrän D 2017 Modelling biodiesel production within a regional context—a comparison with RED Benchmark. Renew. Energy 108, 355–370. (10.1016/j.renene.2017.02.024)

[110] Cerri CEP et al 2017 Assessing the greenhouse gas emissions of Brazilian soybean biodiesel production. PLoS ONE 12, e0176948 (10.1371/journal.pone.0176948) 28493965 PMC5426630

[111] Esteves EMM, Esteves VPP, Bungenstab DJ, Araújo OdQF, Morgado CdRV 2018 Greenhouse gas emissions related to biodiesel from traditional soybean farming compared to integrated crop-livestock systems. J. Clean. Prod. 179, 81–92. (10.1016/j.jclepro.2017.12.262)

[112] Renouf MA, Pagan RJ, Wegener MK 2011 Life cycle assessment of Australian sugarcane products with a focus on cane processing. Int. J. Life Cycle Assess. 16, 125–137. (10.1007/s11367-010-0233-y)

[113] Cox K, Renouf M, Dargan A, Turner C, Klein-Marcuschamer D 2014 Environmental life cycle assessment (LCA) of aviation biofuel from microalgae, *Pongamia pinnata*, and sugarcane molasses. Biofuels Bioprod. Biorefin.-Biofpr. 8, 579–593. (10.1002/bbb.1488)

[114] Gabisa EW, Bessou C, Gheewala SH 2019 Life cycle environmental performance and energy balance of ethanol production based on sugarcane molasses in Ethiopia. J. Cleaner Prod. 234, 43–53. (10.1016/j.jclepro.2019.06.199)

[115] Rathnayake M, Chaireongsirikul T, Svangariyaskul A, Lawtrakul L, Toochinda P 2018 Process simulation based life cycle assessment for bioethanol production from cassava, cane molasses, and rice straw. J. Clean. Prod. 190, 24–35. (10.1016/j.jclepro.2018.04.152)

[116] Papong S, Rewlay-ngoen C, Itsubo N, Malakul P 2017 Environmental life cycle assessment and social impacts of bioethanol production in Thailand. J. Clean. Prod. 157, 254–266. (10.1016/j.jclepro.2017.04.122)

[117] Silalertruksa T, Gheewala SH 2013 A comparative LCA of rice straw utilization for fuels and fertilizer in Thailand. Bioresour. Technol. 150, 412–419. (10.1016/j.biortech.2013.09.015) 24076147

[118] Khatiwada D, Silveira S 2011 Greenhouse gas balances of molasses based ethanol in Nepal. J. Clean. Prod. 19, 1471–1485. (10.1016/j.jclepro.2011.04.012)

[119] Tonini D, Hamelin L, Alvarado-Morales M, Astrup TF 2015 GHG emission factors for bioelectricity, biomethane, and bioethanol quantified for 24 biomass substrates with consequential life-cycle assessment. Bioresour. Technol. 208, 123–133. (10.1016/j.biortech.2016.02.052) 26938807

[120] Zhang Y, Kendall A 2017 Life cycle performance of cellulosic ethanol and corn ethanol from a retrofitted dry mill corn ethanol plant. Bioenergy Res. 2017, 183–198. (10.1007/s12155-016-9776-5)

[121] Panichelli L, Dauriat A, Gnansounou E 2009 Life cycle assessment of soybean-based biodiesel in Argentina for export. Int. J. Life Cycle Assess. 14, 144–159. (10.1007/s11367-008-0050-8)

[122] Dunn JB, Mueller S, Kwon H-Y, Wang MQ 2013 Land-use change and greenhouse gas emissions from corn and cellulosic ethanol. Biotechnol. Biofuels 6, 51 (10.1186/1754-6834-6-51) 23575438 PMC3662634

[123] Tonini D, Astrup T 2012 LCA of biomass-based energy systems: a case study for Denmark. Appl. Energy 99, 234–246. (10.1016/j.apenergy.2012.03.006)

[124] Reijnders L, Huijbregts MAJ 2008 Palm oil and the emission of carbon-based greenhouse gases. J. Clean. Prod. 16, 477–482. (10.1016/j.jclepro.2006.07.054)

[125] Harding KG, Dennis JS, von Blottnitz H, Harrison STL 2008 A life-cycle comparison between inorganic and biological catalysis for the production of biodiesel. J. Clean. Prod. 16, 1368–1378. (10.1016/j.jclepro.2007.07.003)

[126] Fernández-Tirado F, Parra-López C, Romero-Gámez M 2016 Life cycle assessment of biodiesel in Spain: comparing the environmental sustainability of Spanish production versus Argentinean imports. Energy Sustain. Dev. 33, 36–52. (10.1016/j.esd.2016.04.005)

[127] Hansen S 2007 Feasibility study of performing an life cycle assessment on crude palm oil production in Malaysia. Int. J. Life Cycle Assess. 12, 50–58. (10.1065/lca2005.08.226)

[128] Guo M, Littlewood J, Joyce J, Murphy R 2014 The environmental profile of bioethanol produced from current and potential future poplar feedstocks in the EU. Green Chem. 16, 4680–4695. (10.1039/C4GC01124D)

[129] Wang L, Littlewood J, Murphy RJ 2013 Environmental sustainability of bioethanol production from wheat straw in the UK. Renew. Sustain. Energy Rev. 28, 715–725. (10.1016/j.rser.2013.08.031)

[130] Zhao Y, Damgaard A, Xu Y, Liu S, Christensen TH 2019 Bioethanol from corn stover—global warming footprint of alternative biotechnologies. Appl. Energy. 247, 237–253. (10.1016/j.apenergy.2019.04.037)

[131] Liu C, Huang Y, Wang X, Tai Y, Liu L, Liu H 2018 Total environmental impacts of biofuels from corn stover using a hybrid life cycle assessment model combining process life cycle assessment and economic input–output life cycle assessment. Integr. Environ. Assess. Manag. 14, 139–149. (10.1002/ieam.1969) 28796442

[132] Jeswani HK, Falano T, Azapagic A 2015 Life cycle environmental sustainability of lignocellulosic ethanol produced in integrated thermo-chemical biorefineries. Biofuels Bioprod. Biorefin.-Biofpr. 9, 661–676. (10.1002/bbb.1558)

[133] Budsberg E, Crawford J, Gustafson R, Bura R, Puettmann M 2015 Ethanologens vs. acetogens: environmental impacts of two ethanol fermentation pathways. BiomassBioenergy 83, 23–31. (10.1016/j.biombioe.2015.08.019)

[134] Brynolf S, Fridell E, Andersson K 2014 Environmental assessment of marine fuels: liquefied natural gas, liquefied biogas, methanol and bio-methanol. J. Clean. Prod. 74, 86–95. (10.1016/j.jclepro.2014.03.052)

[135] Guo M, Li C, Facciotto G, Bergante S, Bhatia R, Comolli R, Ferre C, Murphy R 2015 Bioethanol from poplar clone Imola: an environmentally viable alternative to fossil fuel? Biotechnol. Biofuels 8, Article number: 134. 10.1186/s13068-015-0318-8 PMC4558961 26339291

[136] Falano T, Jeswani HK, Azapagic A 2014 Assessing the environmental sustainability of ethanol from integrated biorefineries. Biotechnol. J. 9, 753–765. (10.1002/biot.201300246) 24478110 PMC4674963

[137] Reyes VC, Villanueva PAL, Vidal-Barrero F, Ollero P 2015 Integrated economic and life cycle assessment of thermochemical production of bioethanol to reduce production cost by exploiting excess of greenhouse gas savings. Appl. Energy. 148, 466–475. (10.1016/j.apenergy.2015.03.113)

[138] Iribarren D, Peters JF, Dufour J 2012 Life cycle assessment of transportation fuels from biomass pyrolysis. Fuel 97, 812–821. (10.1016/j.fuel.2012.02.053)

[139] González-García S, Iribarren D, Susmozas A, Dufour J, Murphy RJ 2012 Life cycle assessment of two alternative bioenergy systems involving *Salix* spp. biomass: bioethanol production and power generation. Appl. Energy. 95, 111–122. (10.1016/j.apenergy.2012.02.022)

[140] Weinberg J, Kaltschmitt M 2013 Life cycle assessment of mobility options using wood based fuels—comparison of selected environmental effects and costs. Bioresour. Technol. 150, 420–428. (10.1016/j.biortech.2013.08.093) 24012134

[141] Maleche E, Glaser R, Marker T, Shonnard D 2014 A preliminary life cycle assessment of biofuels produced by the IH2 (TM) process. Environ. Prog. Sustain. Energy 33, 322–329. (10.1002/ep.11773)

[142] Slade R, Bauen A, Shah N 2009 The greenhouse gas emissions performance of cellulosic ethanol supply chains in Europe. Biotechnol. Biofuels 2, 15 (10.1186/1754-6834-2-15) 19682352 PMC2746206

[143] Daystar J, Reeb C, Gonzalez R, Venditti R, Kelley SS 2015 Environmental life cycle impacts of cellulosic ethanol in the Southern US produced from loblolly pine, eucalyptus, unmanaged hardwoods, forest residues, and switchgrass using a thermochemical conversion pathway. Fuel Process. Technol. 138: 164–174. (10.1016/j.fuproc.2015.04.019)

[144] Zaimes GG, Soratana K, Harden CL, Landis AE, Khanna V 2015 Biofuels via fast pyrolysis of perennial grasses: a life cycle evaluation of energy consumption and greenhouse gas emissions. Environ. Sci. Technol. 49, 10 007–10 018. (10.1021/acs.est.5b00129) 26196154

[145] Scown CD et al 2012 Lifecycle greenhouse gas implications of US national scenarios for cellulosic ethanol production. Environ. Res. Lett. 7, 014011 (10.1088/1748-9326/7/1/014011)

[146] Choudhary S, Liang S, Cai H, Keoleian GA, Miller SA, Kelly J, Xu M 2014 Reference and functional unit can change bioenergy pathway choices. Int. J. Life Cycle Assess. 19, 796–805. (10.1007/s11367-013-0692-z)

[147] Sinistore JC, Reinemann DJ, Izaurralde RC, Cronin KR, Meier PJ, Runge TM, Zhang X 2015 Life cycle assessment of switchgrass cellulosic ethanol production in the Wisconsin and Michigan agricultural contexts. Bioenergy Res. 8, 897–909. (10.1007/s12155-015-9611-4)

[148] Bai Y, Luo L, van der Voet E 2010 Life cycle assessment of switchgrass-derived ethanol as transport fuel. Int. J. Life Cycle Assess. 15, 468–477. (10.1007/s11367-010-0177-2)

[149] Argo AM et al 2013 Investigation of biochemical biorefinery sizing and environmental sustainability impacts for conventional bale system and advanced uniform biomass logistics designs. Biofuels Bioprod. Biorefin.-Biofpr. 7, 282–302. (10.1002/bbb.1391)

[150] Patrizi N, Caro D, Pulselli FM, Bjerre AB, Bastianoni S 2013 Environmental feasibility of partial substitution of gasoline with ethanol in the Province of Siena (Italy). J. Clean. Prod. 47, 388–395. (10.1016/j.jclepro.2012.11.023)

[151] Moller F, Slento E, Frederiksen P 2014 Integrated well-to-wheel assessment of biofuels combining energy and emission LCA and welfare economic cost benefit analysis. Biomass Bioenergy 60, 41–49. (10.1016/j.biombioe.2013.11.001)

[152] Peters JF, Iribarren D, Dufour J 2015 Simulation and life cycle assessment of biofuel production via fast pyrolysis and hydroupgrading. Fuel 139, 441–456. (10.1016/j.fuel.2014.09.014)

[153] Kumar D, Murthy GS 2012 Life cycle assessment of energy and GHG emissions during ethanol production from grass straws using various pretreatment processes. Int. J. Life Cycle Assess. 17, 388–401. (10.1007/s11367-011-0376-5)

[154] Dang Q, Yu C, Luo Z 2014 Environmental life cycle assessment of bio-fuel production via fast pyrolysis of corn stover and hydroprocessing. Fuel 131, 36–42. (10.1016/j.fuel.2014.04.029)

[155] Nguyen L, Cafferty KG, Searcy EM, Spatari S 2014 Uncertainties in life cycle greenhouse gas emissions from advanced biomass feedstock logistics supply chains in Kansas. Energies 7, 7125–7146. (10.3390/en7117125)

[156] Palma-Rojas S, Caldeira-Pires A, Nogueira JM 2017 Environmental and economic hybrid life cycle assessment of bagasse-derived ethanol produced in Brazil. Int. J. Life Cycle Assess. 22, 317–327. (10.1007/s11367-015-0892-9)

[157] Wang H, Zhang S, Bi X, Clift R 2020 Greenhouse gas emission reduction potential and cost of bioenergy in British Columbia, Canada. Energy Policy 138, 111285 (10.1016/j.enpol.2020.111285)

[158] Haus S, Björnsson L, Börjesson P 2020 Lignocellulosic ethanol in a greenhouse gas emission reduction obligation system—a case study of Swedish sawdust based-ethanol production. Energies 13, 1048 (10.3390/en13051048)

[159] Zupko R 2019 Life cycle assessment of the production of gasoline and diesel from forest residues using integrated hydropyrolysis and hydroconversion. Int. J. Life Cycle Assess. 24, 1793–1804. (10.1007/s11367-019-01616-8)

[160] Lask J, Wagner M, Trindade LM, Lewandowski I 2019 Life cycle assessment of ethanol production from miscanthus: a comparison of production pathways at two European sites. GCB Bioenergy 11, 269–288. (10.1111/gcbb.12551)

[161] Gonzalez-Garcia S, Teresa Moreira M, Feijoo G 2010 Comparative environmental performance of lignocellulosic ethanol from different feedstocks. Renew. Sustain. Energy Rev. 14, 2077–2085. (10.1016/j.rser.2010.03.035)

[162] Parajuli R, Knudsen MT, Birkved M, Djomo SN, Corona A, Dalgaard T 2017 Environmental impacts of producing bioethanol and biobased lactic acid from standalone and integrated biorefineries using a consequential and an attributional life cycle assessment approach. Sci. Total Environ. 598, 497–512. (10.1016/j.scitotenv.2017.04.087) 28448939

[163] Obnamia JA, Dias GM, MacLean HL, Saville BA 2019 Comparison of U.S. Midwest corn stover ethanol greenhouse gas emissions from GREET and GHGenius. Appl. Energy 235, 591–601. (10.1016/j.apenergy.2018.10.091)

[164] Eshton B, Katima JHY, Kituyi E 2013 Greenhouse gas emissions and energy balances of jatropha biodiesel as an alternative fuel in Tanzania. Biomass Bioenergy 58, 95–103. (10.1016/j.biombioe.2013.08.020)

[165] Hagman J, Nerentorp M, Arvidsson R, Molander S 2013 Do biofuels require more water than do fossil fuels? Life cycle-based assessment of jatropha oil production in rural Mozambique. J. Clean. Prod. 53, 176–185. (10.1016/j.jclepro.2013.03.039)

[166] Ndong R, Montrejaud-Vignoles M, Saint Girons O, Gabrielle B, Pirot R, Domergue M, Sablayrolles C 2009 Life cycle assessment of biofuels from *Jatropha curcas* in West Africa: a field study. GCB Bioenergy 1, 197–210. (10.1111/j.1757-1707.2009.01014.x)

[167] Ajayebi A, Gnansounou E, Raman JK 2013 Comparative life cycle assessment of biodiesel from algae and jatropha: a case study of India. Bioresour. Technol. 150, 429–437. (10.1016/j.biortech.2013.09.118) 24140355

[168] Kumar S, Singh J, Nanoti SM, Garg MO 2012 A comprehensive life cycle assessment (LCA) of Jatropha biodiesel production in India. Bioresour. Technol. 110, 723–729. (10.1016/j.biortech.2012.01.142) 22361070

[169] Li X, Mupondwa E 2014 Life cycle assessment of camelina oil derived biodiesel and jet fuel in the Canadian Prairies. Sci. Total Environ. 481, 17–26. (10.1016/j.scitotenv.2014.02.003) 24572928

[170] Krohn BJ, Fripp M 2012 A life cycle assessment of biodiesel derived from the ‘niche filling’ energy crop camelina in the USA. Appl. Energy. 92, 92–98. (10.1016/j.apenergy.2011.10.025)

[171] Escobar N, Ribal J, Clemente G, Sanjuan N 2014 Consequential LCA of two alternative systems for biodiesel consumption in Spain, considering uncertainty. J. Clean. Prod. 79, 61–73. (10.1016/j.jclepro.2014.05.065)

[172] Caldeira C, Queirós J, Noshadravan A, Freire F 2016 Incorporating uncertainty in the life cycle assessment of biodiesel from waste cooking oil addressing different collection systems. Resour. Conserv. Recycling 112, 83–92. (10.1016/j.resconrec.2016.05.005)

[173] Talens PL, Lombardi L, Villalba Méndez G, Gabarrell i Durany X 2010 Life cycle assessment (LCA) and exergetic life cycle assessment (ELCA) of the production of biodiesel from used cooking oil (UCO). Energy 35, 889–893. (10.1016/j.energy.2009.07.013)

[174] Dufour J, Iribarren D 2012 Life cycle assessment of biodiesel production from free fatty acid-rich wastes. Renew. Energy 38, 155–162. (10.1016/j.renene.2011.07.016)

[175] Thamsiriroj T, Murphy JD 2011 The impact of the life cycle analysis methodology on whether biodiesel produced from residues can meet the EU sustainability criteria for biofuel facilities constructed after 2017. Renew. Energy 36, 50–63. (10.1016/j.renene.2010.05.018)

[176] Souza DdP, Mendonca FM, Alves Nunes KR, Valle R 2012 Environmental and socioeconomic analysis of producing biodiesel from used cooking oil in Rio de Janeiro: the case of the Copacabana District. J. Ind. Ecol. 16, 655–664. (10.1111/j.1530-9290.2012.00517.x)

[177] Pleanjai S, Gheewala SH, Garivait S 2009 Greenhouse gas emissions from production and use of used cooking oil methyl ester as transport fuel in Thailand. J. Clean. Prod. 17, 873–876. (10.1016/j.jclepro.2009.01.007)

[178] Mortimer N, Evans AKF, Mwabonje O, Whittaker CL, Hunter AJ 2010 Comparison of the greenhouse gas benefits resulting from use of vegetable oils for electricity, heat, transport and industrial purposes. NNFCC.

[179] Vrech A, Ferfuia C, Bessong Ojong W, Piasentier E, Baldini M 2019 Energy and environmental sustainability of Jatropha-Biofuels Chain from nontoxic accessions in Cameroon. Environ. Prog. Sustain. Energy 38, 305–314. (10.1002/ep.12928)

[180] Fuentes A, García C, Hennecke A, Masera O 2018 Life cycle assessment of *Jatropha curcas* biodiesel production: a case study in Mexico. Clean Technol. Environ. Policy 20, 1721–1733. (10.1007/s10098-018-1558-7)

[181] Baumert S, Khamzina A, Vlek PLG 2018 Greenhouse gas and energy balance of Jatropha biofuel production systems of Burkina Faso. Energy Sustain. Dev. 42, 14–23. (10.1016/j.esd.2017.09.007)

[182] Khang DS, Tan RR, Uy OM, Promentilla MAB, Tuan PD, Abe N, Razon LF 2017 Design of experiments for global sensitivity analysis in life cycle assessment: the case of biodiesel in Vietnam. Resour. Conserv. Recycl. 119, 12–23. (10.1016/j.resconrec.2016.08.016)

[183] Giraldi-Díaz MR, De Medina-Salas L, Castillo-González E, De la Cruz-Benavides M 2018 Environmental impact associated with the supply chain and production of biodiesel from *Jatropha curcas* L. through life cycle analysis. Sustainability 10, 1451 (10.3390/su10051451)

[184] Bacenetti J, Restuccia A, Schillaci G, Failla S 2017 Biodiesel production from unconventional oilseed crops (*Linum usitatissimum* L. and *Camelina sativa* L.) in Mediterranean conditions: environmental sustainability assessment. Renew. Energy 112, 444–456. (10.1016/j.renene.2017.05.044)

[185] Tabatabaie SMH, Tahami H, Murthy GS 2018 A regional life cycle assessment and economic analysis of camelina biodiesel production in the Pacific Northwestern US. J. Clean. Prod. 172, 2389–2400. (10.1016/j.jclepro.2017.11.172)

[186] Foteinis S, Chatzisymeon E, Litinas A, Tsoutsos T 2020 Used-cooking-oil biodiesel: life cycle assessment and comparison with first- and third-generation biofuel. Renew. Energy 153, 588–600. (10.1016/j.renene.2020.02.022)

[187] Faleh N, Khila Z, Wahada Z, Pons M-N, Houas A, Hajjaji N 2018 Exergo-environmental life cycle assessment of biodiesel production from mutton tallow transesterification. Renew. Energy. 127, 74–83. (10.1016/j.renene.2018.04.046)

[188] Lombardi L, Mendecka B, Carnevale E 2018 Comparative life cycle assessment of alternative strategies for energy recovery from used cooking oil. J. Environ. Manage. 216, 235–245. (10.1016/j.jenvman.2017.05.016) 28521956

[189] Yang Y, Fu T, Bao W, Xie GH 2017 Life cycle analysis of greenhouse gas and PM2.5 emissions from restaurant waste oil used for biodiesel production in China. BioEnergy Res. 10, 199–207. (10.1007/s12155-016-9792-5)

[190] Mu D, Min M, Krohn B, Mullins KA, Ruan R, Hill J 2014 Life cycle environmental impacts of wastewater-based algal biofuels. Environ. Sci. Technol. 48, 11 696–11 704. (10.1021/es5027689) 25220843

[191] Pragya N, Pandey KK 2016 Life cycle assessment of green diesel production from microalgae. Renew. Energy 86, 623–632. (10.1016/j.renene.2015.08.064)

[192] Yuan J, Kendall A, Zhang Y 2015 Mass balance and life cycle assessment of biodiesel from microalgae incorporated with nutrient recycling options and technology uncertainties. Global Change Biol. Bioenergy. 7, 1245–1259. (10.1111/gcbb.12229)

[193] Soratana K, Harper WF Jr, Landis AE 2012 Microalgal biodiesel and the renewable fuel standard's greenhouse gas requirement. Energy Policy 46, 498–510. (10.1016/j.enpol.2012.04.016)

[194] Chowdhurya R, Freire F 2015 Bioenergy production from algae using dairy manure as a nutrient source: life cycle energy and greenhouse gas emission analysis. Appl. Energy. 154, 1112–1121. (10.1016/j.apenergy.2015.05.045)

[195] Medeiros DL, Sales EA, Kiperstok A 2015 Energy production from microalgae biomass: carbon footprint and energy balance. J. Clean. Prod. 96, 493–500. (10.1016/j.jclepro.2014.07.038)

[196] Adesanya VO, Cadena E, Scott SA, Smith AG 2014 Life cycle assessment on microalgal biodiesel production using a hybrid cultivation system. Bioresour. Technol. 163, 343–355. (10.1016/j.biortech.2014.04.051) 24852435

[197] Sills DL, Paramita V, Franke MJ, Johnson MC, Akabas TM, Greene CH, Tester JW 2013 Quantitative uncertainty analysis of life cycle assessment for algal biofuel production. Environ. Sci. Technol. 47, 687–694. (10.1021/es3029236) 23237457

[198] Campbell PK, Beer T, Batten D 2011 Life cycle assessment of biodiesel production from microalgae in ponds. Bioresour. Technol. 102, 50–56. (10.1016/j.biortech.2010.06.048) 20594828

[199] Stephenson AL, Kazamia E, Dennis JS, Howe CJ, Scott SA, Smith AG 2010 Life-cycle assessment of potential algal biodiesel production in the United Kingdom: a comparison of raceways and air-lift tubular bioreactors. Energy Fuels 24, 4062–4077. (10.1021/ef1003123)

[200] Sander K, Murthy GS 2010 Life cycle analysis of algae biodiesel. Int. J. Life Cycle Assess. 15, 704–714. (10.1007/s11367-010-0194-1)

[201] Holma A, Koponen K, Antikainen R, Lardon L, Leskinen P, Roux P 2013 Current limits of life cycle assessment framework in evaluating environmental sustainability—case of two evolving biofuel technologies. J. Clean. Prod. 54, 215–228. (10.1016/j.jclepro.2013.04.032)

[202] Woertz IC, Benemann JR, Du N, Unnasch S, Mendola D, Mitchell BG, Lundquist TJ 2014 Life cycle GHG emissions from microalgal biodiesel—a CA-GREET model. Environ. Sci. Technol. 48, 6060–6068. (10.1021/es403768q) 24779347

[203] Bennion EP, Ginosar DM, Moses J, Agblevor F, Quinn JC 2015 Lifecycle assessment of microalgae to biofuel: comparison of thermochemical processing pathways. Appl. Energy. 154, 1062–1071. (10.1016/j.apenergy.2014.12.009)

[204] Azari A, Noorpoor AR, Bozorg-Haddad O 2019 Carbon footprint analyses of microalgae cultivation systems under autotrophic and heterotrophic conditions. Int. J. Environ. Sci. Technol. 16, 6671–6684. (10.1007/s13762-018-2072-5)

[205] Morales M, Hélias A, Bernard O 2019 Optimal integration of microalgae production with photovoltaic panels: environmental impacts and energy balance. Biotechnol. Biofuels 12, 239 (10.1186/s13068-019-1579-4) 31624501 PMC6781331

[206] Sun C-H, Fu Q, Liao Q, Xia A, Huang Y, Zhu X, Reugsang A, Chang H-X 2019 Life-cycle assessment of biofuel production from microalgae via various bioenergy conversion systems. Energy 171, 1033–1045. (10.1016/j.energy.2019.01.074)

[207] Wu W, Lin K-H, Chang J-S 2018 Economic and life-cycle greenhouse gas optimization of microalgae-to-biofuels chains. Bioresour. Technol. 267, 550–559. (10.1016/j.biortech.2018.07.083) 30053713

[208] Foteinis S, Antoniadis-Gavriil A, Tsoutsos T 2018 Life cycle assessment of algae-to-biodiesel shallow pond production systems in the Mediterranean: influence of species, pond type, by(co)-product valorisation and electricity mix. Biofuels Bioprod. Biorefin. 12, 542–558. (10.1002/bbb.1871)

[209] Bello M, Ranganathan P, Brennan F 2017 Life cycle optimization for sustainable algal biofuel production using integrated nutrient recycling technology. ACS Sustain. Chem. Eng. 5, 9869–9880. (10.1021/acssuschemeng.7b01833)

[210] Lisboa CC, Butterbach-Bahl K, Mauder M, Kiese R 2011 Bioethanol production from sugarcane and emissions of greenhouse gases—known and unknowns. Global Change Biol. Bioenergy 3, 277–292. (10.1111/j.1757-1707.2011.01095.x)

[211] Esteves VPP et al 2016 Land use change (LUC) analysis and life cycle assessment (LCA) of Brazilian soybean biodiesel. Clean Technol. Environ. Policy. 18, 1655–1673. (10.1007/s10098-016-1161-8)

[212] Ecofys. 2015 The land use change impact of biofuels consumed in the EU quantification of area and greenhouse gas impacts. Utrecht, The Netherlands: Ecofys.

[213] Repo A, Böttcher H, Kindermann G, Liski J 2015 Sustainability of forest bioenergy in Europe: land-use-related carbon dioxide emissions of forest harvest residues. GCB Bioenergy 7, 877–887. (10.1111/gcbb.12179)

[214] Warren RD, Bogdanski A, Tittonell P 2015 How does crop residue removal affect soil organic carbon and yield? A hierarchical analysis of management and environmental factors. Biomass Bioenergy. 81, 345–355. (10.1016/j.biombioe.2015.07.022)

[215] Zhao G, Bryan BA, King D, Luo Z, Wang E, Yu Q 2015 Sustainable limits to crop residue harvest for bioenergy: maintaining soil carbon in Australia's agricultural lands. GCB Bioenergy 7, 479–487. (10.1111/gcbb.12145)

[216] Singh A, Olsen SI 2013 Comparison of algal biodiesel production pathways using life cycle assessment tool. In Life cycle assessment of renewable energy sources (eds Singh A, Pant D, Olsen SI), pp. 145–168. London, UK: Springer.

[217] Orfield ND, Levine RB, Keoleian GA, Miller SA, Savage PE 2015 Growing algae for biodiesel on direct sunlight or sugars: a comparative life cycle assessment. Acs Sustain. Chem. Eng. 3, 386–395. (10.1021/sc5004117)

[218] Arvidsson R, Fransson K, Froling M, Svanstrom M, Molander S 2012 Energy use indicators in energy and life cycle assessments of biofuels: review and recommendations. J. Clean. Prod. 31, 54–61. (10.1016/j.jclepro.2012.03.001)

[219] Ponnusamy S, Reddy HK, Muppaneni T, Downes CM, Deng S 2014 Life cycle assessment of biodiesel production from algal bio-crude oils extracted under subcritical water conditions. Bioresour. Technol. 170, 454–461. (10.1016/j.biortech.2014.07.072) 25164337

[220] Pardo-Cardenas Y, Herrera-Orozco I, Gonzalez-Delgado A-D, Kafarov V 2013 Environmental assessment of microalgae biodiesel production in Colombia: comparison of three oil extraction systems. Ct&F-Cienc. Tecn. Fut. 5, 85–100. (10.29047/01225383.59)

[221] Scacchi CCO, Gonzalez-Garcia S, Caserini S, Rigamonti L 2010 Greenhouse gases emissions and energy use of wheat grain-based bioethanol fuel blends. Sci. Total Environ. 408, 5010–5018. (10.1016/j.scitotenv.2010.07.046) 20692687

[222] Kamahara H, Hasanudin U, Widiyanto A, Tachibana R, Atsuta Y, Goto N, Daimon H, Fujie K 2010 Improvement potential for net energy balance of biodiesel derived from palm oil: a case study from Indonesian practice. Biomass Bioenergy 34, 1818–1824. (10.1016/j.biombioe.2010.07.014)

[223] Queiroz AG, Franca L, Ponte MX 2012 The life cycle assessment of biodiesel from palm oil (‘dende’) in the Amazon. Biomass Bioenergy 36, 50–59. (10.1016/j.biombioe.2011.10.007)

[224] Zaimes GG, Khanna V 2014 Assessing the critical role of ecological goods and services in microalgal biofuel life cycles. Rsc Advances. 4, 44 980–44 990. (10.1039/C4RA09191D)

[225] Slade R, Bauen A 2013 Micro-algae cultivation for biofuels: cost, energy balance, environmental impacts and future prospects. Biomass Bioenergy 53, 29–38. (10.1016/j.biombioe.2012.12.019)

[226] National Research Council. 2012 Sustainable development of algal biofuels in the United States. Washington, DC: The National Academies Press.

[227] Dominguez-Faus R, Powers SE, Burken JG, Alvarez PJ 2009 The water footprint of biofuels: a drink or drive issue? Environ. Sci. Technol. 43, 3005–3010. (10.1021/es802162x) 19534106

[228] National Research Council. 2008 Water implications of biofuels production in the United States. Washington, DC: The National Academies Press.

[229] Hammond GP, Li B 2016 Environmental and resource burdens associated with world biofuel production out to 2050: footprint components from carbon emissions and land use to waste arisings and water consumption. GCB Bioenergy 8, 894–908. (10.1111/gcbb.12300) 27610203 PMC4988122

[230] Jeswani HK, Azapagic A 2011 Water footprint: methodologies and a case study for assessing the impacts of water use. J. Clean. Prod. 19, 1288–1299. (10.1016/j.jclepro.2011.04.003)

[231] Gerbens-Leenes W, Hoekstra AY, van der Meer TH 2009 The water footprint of bioenergy. Proc. Natl Acad. Sci. USA 106, 10 219–10 223. (10.1073/pnas.0812619106) 19497862 PMC2690604

[232] Berger M, Pfister S, Bach V, Finkbeiner M 2015 Saving the planet's climate or water resources? The trade-off between carbon and water footprints of European biofuels. Sustainability 7, 6665–6683.

[233] Gerbens-Leenes PW, Xu L, de Vries GJ, Hoekstra AY 2014 The blue water footprint and land use of biofuels from algae. Water Resour. Res. 50, 8549–8563. (10.1002/2014WR015710)

[234] Webb A, Coates D 2012 Biofuels and biodiversity. Montreal, Technical Series No. 65, 69 p. Secretariat of the Convention on Biological Diversity.

[235] Correa DF, Beyer HL, Possingham HP, Thomas-Hall SR, Schenk PM 2017 Biodiversity impacts of bioenergy production: microalgae vs. first generation biofuels. Renew. Sustain. Energy Rev. 74, 1131–1146. (10.1016/j.rser.2017.02.068)

[236] Liu Y, Xu Y, Zhang F, Yun J, Shen Z 2014 The impact of biofuel plantation on biodiversity: a review. Chin. Sci. Bull. 59, 4639–4651. (10.1007/s11434-014-0639-1)

[237] Elshout PMF, van Zelm R, van der Velde M, Steinmann Z, Huijbregts MAJ 2019 Global relative species loss due to first-generation biofuel production for the transport sector. GCB Bioenergy 11, 763–772. (10.1111/gcbb.12597) 31423154 PMC6686982

[238] IEA. 2010 Sustainable production of second-generation biofuels—potential and perspectives in major economies and developing countries. Paris, France: International Energy Agency.

[239] Rowe RL, Street NR, Taylor G 2009 Identifying potential environmental impacts of large-scale deployment of dedicated bioenergy crops in the UK. Renew. Sustain. Energy Rev. 13, 271–290. (10.1016/j.rser.2007.07.008)

[240] The Royal Society. 2008 Sustainable biofuels: prospects and challenges. London, UK: Royal Society.

[241] Mortimer N 2016 Carbon life cycle assessment of bioenergy for policy analysis, formulation and implementation: a briefing paper. North Energy Associates Limited.

[242] Yang Y, Bae J, Kim J, Suh S 2012 Replacing gasoline with corn ethanol results in significant environmental problem-shifting. Environ. Sci. Technol. 46, 3671–3678. (10.1021/es203641p) 22390573

[243] Ripa M, Buonauio C, Melllino S, Fiorentino G, Ulgati S 2014 Recycling waste cooking oil into biodiesel: a life cycle assessment. Int. J. Perform. Eng. 10, 347–356.

[244] Kalaivani K, Ravikumar G, Balasubramanian N 2014 Environmental impact studies of biodiesel production from *Jatropha curcas* in India by life cycle assessment. Environ. Prog. Sustain. Energy 33, 1340–1349. (10.1002/ep.11913)

[245] Whitaker J, Ludley KE, Rowe R, Taylor G, Howard DC 2010 Sources of variability in greenhouse gas and energy balances for biofuel production: a systematic review. GCB Bioenergy 2, 99–112. (10.1111/j.1757-1707.2010.01047.x)

[246] Searchinger T et al 2008 Use of U.S. croplands for biofuels increases greenhouse gases through emissions from land-use change. Science 319, 1238–1240. (10.1126/science.1151861) 18258860

[247] Kim S, Dale BE, Heijungs R, Azapagic A, Darlington T, Kahlbaum D 2014 Indirect land use change and biofuels: mathematical analysis reveals a fundamental flaw in the regulatory approach. Biomass Bioenergy 71, 408–412. (10.1016/j.biombioe.2014.09.015)

[248] Zamagni A, Guinée J, Heijungs R, Masoni P, Raggi A 2012 Lights and shadows in consequential LCA. Int. J. Life Cycle Assess. 17, 904–918. (10.1007/s11367-012-0423-x)

[249] Brander M, Tipper R, Hutchison C, Davis G 2009 *Consequential and attributional approaches to LCA: a guide to policy makers with specific reference to greenhouse gas LCA of biofuels*, p. 14. Edinburgh, UK: Econometrica Press.

[250] Luo L, van der Voet E, Huppes G, Udo de Haes HA 2009 Allocation issues in LCA methodology: a case study of corn stover-based fuel ethanol. Int. J. Life Cycle Assess. 14, 529–539. (10.1007/s11367-009-0112-6)

[251] Cherubini F, Stromman AH 2011 Life cycle assessment of bioenergy systems: state of the art and future challenges. Bioresour. Technol. 102, 437–451. (10.1016/j.biortech.2010.08.010) 20832298

[252] Whitaker J et al 2018 Consensus, uncertainties and challenges for perennial bioenergy crops and land use. GCB Bioenergy 10, 150–164. (10.1111/gcbb.12488) 29497458 PMC5815384

[253] IPCC. 1996 Revised 1996 IPCC guidelines for national greenhouse gas inventories: workbook-module 24 agriculture (eds J Houghton et al.).

[254] Crutzen PJ, Mosier AR, Smith KA, Winiwarter W 2008 N_2_O release from agro-biofuel production negates global warming reduction by replacing fossil fuels. Atmos. Chem. Phys. 8, 289–395. (10.5194/acp-8-389-2008)

[255] Liska AJ 2015 Eight principles of uncertainty for life cycle assessment of biofuel systems. Adam Liska Papers. 26.

[256] Sylvester-Bradley R et al. 2015 Minimising nitrous oxide intensities of arable crop products (MIN-NO) See http://cereals.ahdb.org.uk/media/759084/pr548.pdf.

[257] Farrell AE, Plevin RJ, Turner BT, Jones AD, O'Hare M, Kammen DM 2006 Ethanol can contribute to energy and environmental goals. Science 311, 506–508. (10.1126/science.1121416) 16439656

[258] Humpenoeder F, Schaldach R, Cikovani Y, Schebek L 2013 Effects of land-use change on the carbon balance of 1st generation biofuels: an analysis for the European Union combining spatial modeling and LCA. Biomass Bioenergy 56, 166–178. (10.1016/j.biombioe.2013.05.003)

[259] Yang Y, Suh S 2015 Marginal yield, technological advances, and emissions timing in corn ethanol's carbon payback time. Int. J. Life Cycle Assess. 20, 226–232. (10.1007/s11367-014-0827-x)

[260] Ahlgren S, Di Lucia L 2014 Indirect land use changes of biofuel production—a review of modelling efforts and policy developments in the European Union. Biotechnol. Biofuels 7, 35 (10.1186/1754-6834-7-35) 24602172 PMC4015842

[261] Fritsche UR, Wiegmann K 2011 Indirect land use change and biofuels. European Parliament's Committee on Environment, Public Health and Food Safety.

[262] Plevin RJ, O'Hare M, Jones AD, Torn MS, Gibbs HK 2010 Greenhouse gas emissions from biofuels' indirect land use change are uncertain but may be much greater than previously estimated. Environ. Sci. Technol. 44, 8015–8021. (10.1021/es101946t) 20942480

[263] Lywood W 2010 Issues of concern with models for calculating GHG emissions from indirect land use change, revision 1. Yarm, UK Ensus.

[264] Kline KL, Oladosu GA, Dale VH, McBride AC 2011 Scientific analysis is essential to assess biofuel policy effects: in response to the paper by Kim and Dale on ‘Indirect land-use change for biofuels: Testing predictions and improving analytical methodologies’. Biomass Bioenergy. 35, 4488–4491. (10.1016/j.biombioe.2011.08.011)

[265] Agricultural Industries Confederation (AIC), National Farmers Union (NFU), Renewable Energy Association (REA), the Seed Crushers and Oil Processors Association (SCOPA). 2013 The effects on the UK biofuel industry of the EU proposals on Indirect Land Use Change (COM 2012) 595 final of 17 October 2012, and UK Biofuels Industry counter-proposals See www.r-e-a.net/resources/pdf/104/FINALREANFUSCOPAAICIndustry_positionILUC18Feb13.pdf.

[266] Linares P, Pérez-Arriaga IJ 2013 A sustainable framework for biofuels in Europe. Energy Policy. 52, 166–169. (10.1016/j.enpol.2012.10.008)

[267] Qin Z, Dunn JB, Kwon H, Mueller S, Wander MM 2016 Soil carbon sequestration and land use change associated with biofuel production: empirical evidence. Global Change Biol. Bioenergy 8, 66–80. (10.1111/gcbb.12237)

[268] Smith P 2012 Soils and climate change. Curr. Opin. Environm. Sustain. 4, 539–544. (10.1016/j.cosust.2012.06.005)

[269] Cherubini F, Ulgiati S 2010 Crop residues as raw materials for biorefinery systems—a LCA case study. Appl. Energy. 87, 47–57. (10.1016/j.apenergy.2009.08.024)

[270] Koponen K, Soimakallio S, Tsupari E, Thun R, Antikainen R 2013 GHG emission performance of various liquid transportation biofuels in Finland in accordance with the EU sustainability criteria. Appl. Energy. 102, 440–448. (10.1016/j.apenergy.2012.07.023)

[271] Liska AJ, Yang H, Milner M, Goddard S, Blanco-Canqui H, Pelton MP, Fang XX, Zhu H, Suyker AE 2014 Biofuels from crop residue can reduce soil carbon and increase CO_2_ emissions. Nat. Clim. Change 4, 398–401. (10.1038/nclimate2187)

[272] Whittaker C, Borrion AL, Newnes L, McManus M 2014 The renewable energy directive and cereal residues. Appl. Energy. 122, 207–215. (10.1016/j.apenergy.2014.01.091)

[273] Qin Z, Dunn JB, Kwon H, Mueller S, Wander MM 2016 Influence of spatially dependent, modeled soil carbon emission factors on life-cycle greenhouse gas emissions of corn and cellulosic ethanol. GCB Bioenergy 8, 1136–1149. (10.1111/gcbb.12333)

[274] Rowe RL, Keith AM, Elias D, Dondini M, Smith P, Oxley J, McNamara NP 2016 Initial soil C and land-use history determine soil C sequestration under perennial bioenergy crops. GCB Bioenergy 8, 1046–1060. (10.1111/gcbb.12311)

[275] Richards M et al 2017 High-resolution spatial modelling of greenhouse gas emissions from land-use change to energy crops in the United Kingdom. GCB Bioenergy 9, 627–644. (10.1111/gcbb.12360)

[276] Brandão M, Milà i Canals L, Clift R 2011 Soil organic carbon changes in the cultivation of energy crops: implications for GHG balances and soil quality for use in LCA. Biomass Bioenergy. 35, 2323–2336. (10.1016/j.biombioe.2009.10.019)

[277] Schmer MR, Jin VL, Wienhold BJ 2015 Sub-surface soil carbon changes affects biofuel greenhouse gas emissions. Biomass Bioenergy 81, 31–34. (10.1016/j.biombioe.2015.05.011)

[278] Goglio P, Smith WN, Grant BB, Desjardins RL, McConkey BG, Campbell CA, Nemecek T 2015 Accounting for soil carbon changes in agricultural life cycle assessment (LCA): a review. J. Clean. Prod. 104, 23–39. (10.1016/j.jclepro.2015.05.040)

[279] International Council on Clean Transportation. 2014 Comprehensive carbon accounting for identification of sustainable biomass feedstocks. Washington, DC: The International Council on Clean Transportation.

[280] Cherubini F, Peters GP, Berntsen T, Strømman AH, Hertwich E 2011 CO_2_ emissions from biomass combustion for bioenergy: atmospheric decay and contribution to global warming. GCB Bioenergy 3, 413–426. (10.1111/j.1757-1707.2011.01102.x)

[281] World Resource Institute, World Business Council for Sustainable Development. 2011 Product life cycle accounting and reporting standard—greenhouse gas protocol. USA: World Resource Institute and World Business Council for Sustainable Development See https://ghgprotocol.org/sites/default/files/standards/Product-Life-Cycle-Accounting-Reporting-Standard_041613.pdf.

[282] BSI. 2011 Publicly available specification 2050:2011—specification for the assessment of the life cycle greenhouse gas emissions of goods and services. London, UK: British Standards Institute.

[283] ISO. 2013 ISO 14067—greenhouse gases—carbon footprint of products—requirements and guidelines for quantification and communication. Geneva, Switzerland: International Organization for Standardization.

[284] Scovronick N, Wilkinson P 2014 Health impacts of liquid biofuel production and use: a review. Global Environ. Change 24, 155–164. (10.1016/j.gloenvcha.2013.09.011)

[285] Tessum CW, Marshall JD, Hill JD 2012 A spatially and temporally explicit life cycle inventory of air pollutants from gasoline and ethanol in the United States. Environ. Sci. Technol. 46, 11 408–11 417. (10.1021/es3010514) 22906224

[286] Scovronick N et al 2016 Air auality and health impacts of future ethanol production and use in São Paulo State, Brazil. Int. J. Environ. Res. Public Health. 13, 695 (10.3390/ijerph13070695) PMC4962236 27409628

[287] Le Blond JS, Woskie S, Horwell CJ, Williamson BJ 2017 Particulate matter produced during commercial sugarcane harvesting and processing: a respiratory health hazard? Atmos. Environ. 149, 34–46. (10.1016/j.atmosenv.2016.11.012)

[288] Arbex MA, Pereira LAA, Carvalho-Oliveira R, Saldiva PHdN, Braga ALF 2014 The effect of air pollution on pneumonia-related emergency department visits in a region of extensive sugar cane plantations: a 30-month time-series study. J. Epidemiol. Community Health. 68, 669–674. (10.1136/jech-2013-203709) 24782416

[289] Silveira HCS et al 2013 Emissions generated by sugarcane burning promote genotoxicity in rural workers: a case study in Barretos, Brazil. Environ. Health. 12, 87 (10.1186/1476-069X-12-87) 24112819 PMC4126064

[290] Air Quality Expert Group. 2011 Road transport biofuels: impact on UK air quality. London, UK: DEFRA See https://uk-air.defra.gov.uk/assets/documents/110322_AQEG_Biofuels_advice_note.pdf.

[291] Beer T et al 2011 The health impacts of ethanol blend petrol. Energies 4, 352 (10.3390/en4020352)

[292] Wallington TJ, Anderson JE, Kurtz EM, Tennison PJ 2016 Biofuels, vehicle emissions, and urban air quality. Faraday Discuss. 189, 121–136. (10.1039/C5FD00205B) 27112132

[293] Sundvor I, López-Aparicio S 2014 Impact of bioethanol fuel implementation in transport based on modelled acetaldehyde concentration in the urban environment. Sci. Total Environ. 496, 100–106. (10.1016/j.scitotenv.2014.07.017) 25064718

[294] Rouleau M, Egyed M, Taylor B, Chen J, Samaali M, Davignon D, Morneau G 2013 Human health impacts of biodiesel use in on-road heavy duty diesel vehicles in Canada. Environ. Sci. Technol. 47, 13 113–13 121. (10.1021/es4023859) 24143909

[295] Hutter H-P, Kundi M, Moshammer H, Shelton J, Krüger B, Schicker I, Wallner P 2015 Replacing fossil diesel by biodiesel fuel: expected impact on health. Arch. Environ. Occup. Health. 70, 4–9. (10.1080/19338244.2013.787962) 24965323

[296] Larcombe AN, Kicic A, Mullins BJ, Knothe G 2015 Biodiesel exhaust: the need for a systematic approach to health effects research. Respirology 20, 1034–1045. (10.1111/resp.12587) 26179557

