# Supplementary material for: Environmental sustainability of biofuels: a review
Source: Proc Math Phys Eng Sci. 2020 Nov 25;476(2243):20200351. doi: 10.1098/rspa.2020.0351 (PMC7735313; doi:10.1098/rspa.2020.0351)
Supplement: Detailed data on GHG emissions from different biofuels [file rspa20200351supp1.pdf]

Environmental sustainability of biofuels: A review

Harish K. Jeswani, Andrew Chilvers and Adisa Azapagic

Supporting information

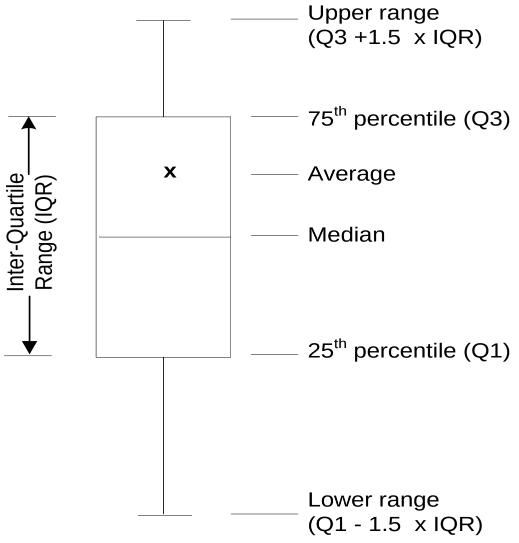

Figure S1: Legend for Figures 2-6

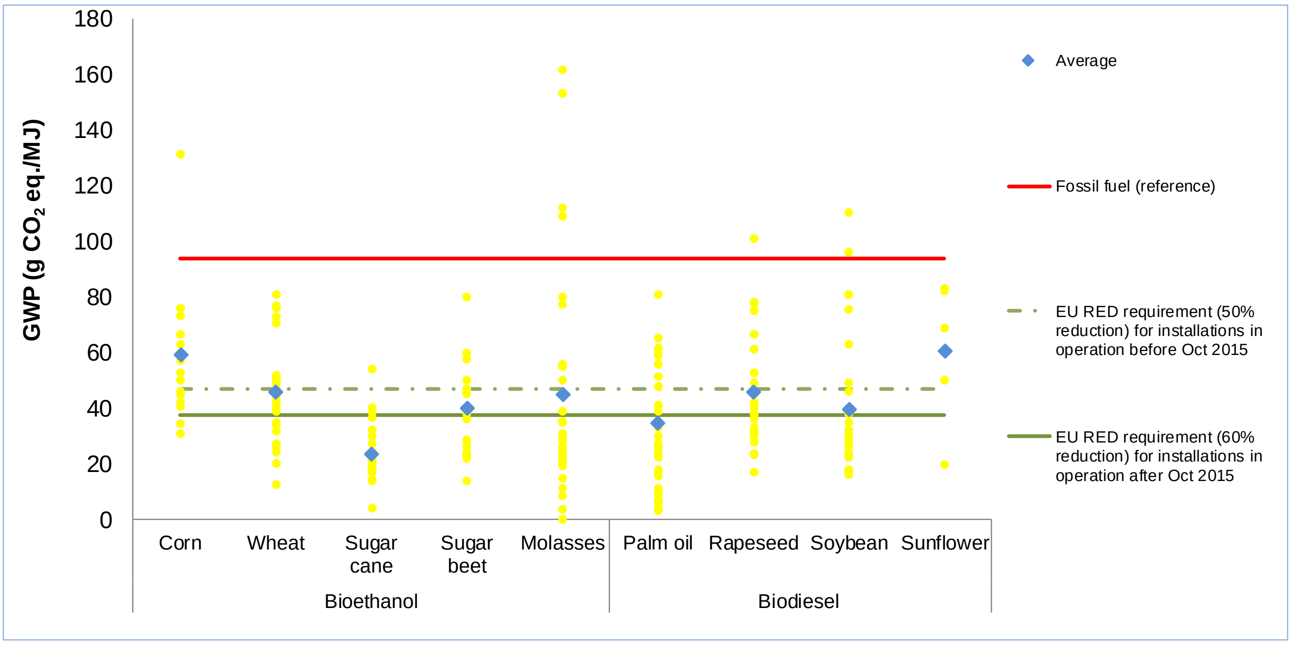

Figure S2: Global warming potential of first generation biofuels (without LUC) reported in LCA studies [1-76]

[The values in this figure were used to generate plots in Figure 2 in the paper.]

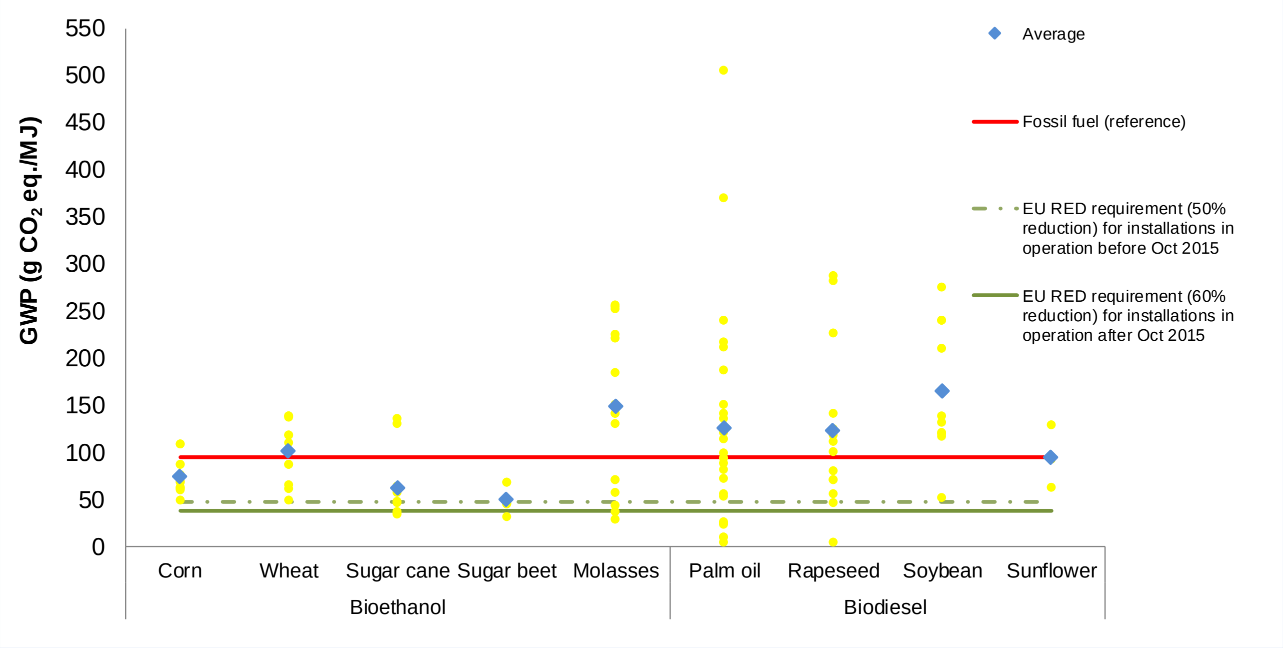

Figure S3: Global warming potential of first generation biofuels (with land use change) reported in LCA studies [1, 3, 10, 11, 16, 22, 23, 29-34, 36, 37, 40, 51, 60, 61, 64, 73, 77-86]
[The values in this figure were used to generate plots in Figure 3 in the paper.]

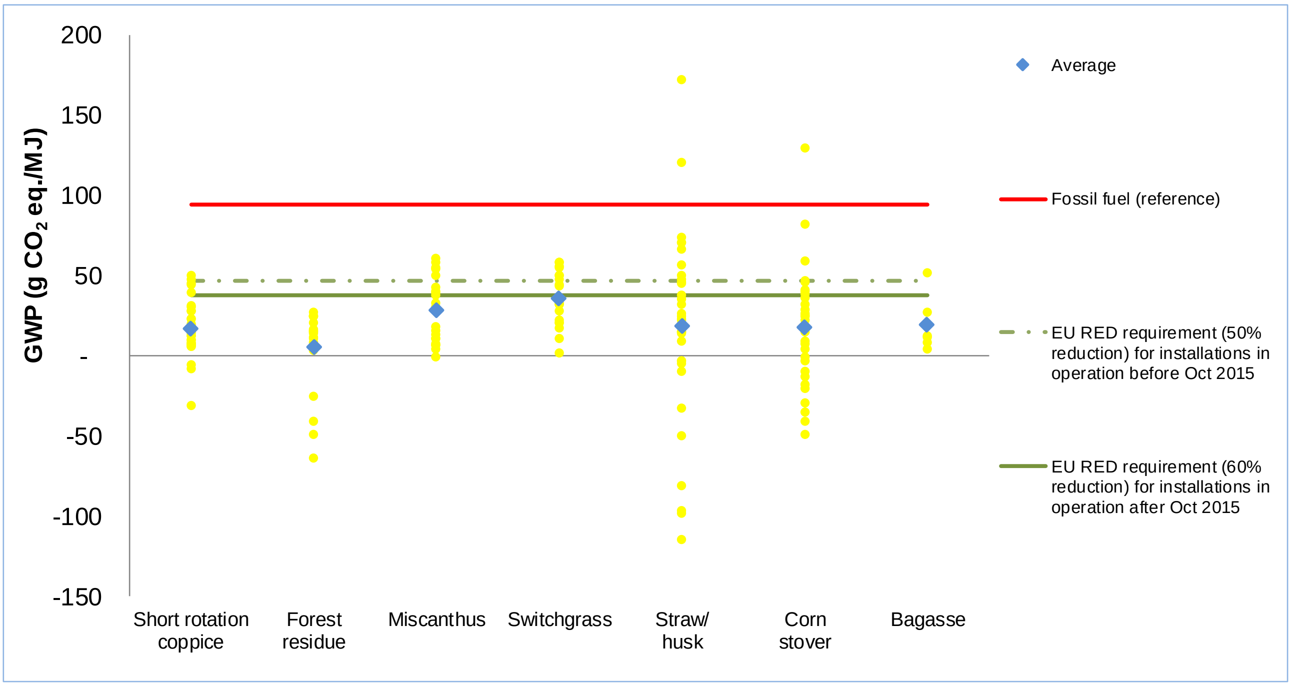

Figure S4: Global warming potential of second generation bioethanol reported in LCA studies [1, 3, 20, 55, 57, 58, 72, 74, 77, 86-126]
[The values in this figure were used to generate plots in Figure 4 in the paper.]

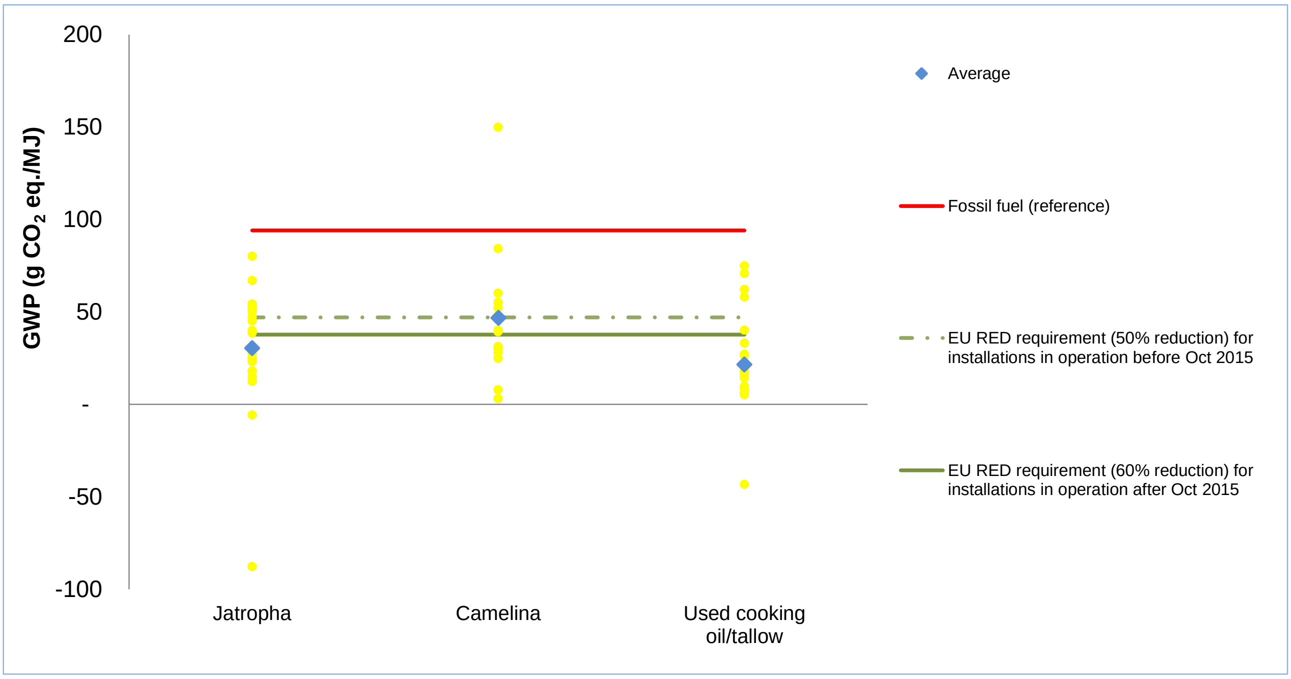

Figure S5: Global warming potential of second generation biodiesel reported in LCA studies [5, 38, 39, 48, 64, 127-153]
[The values in this figure were used to generate plots in Figure 5 in the paper.]

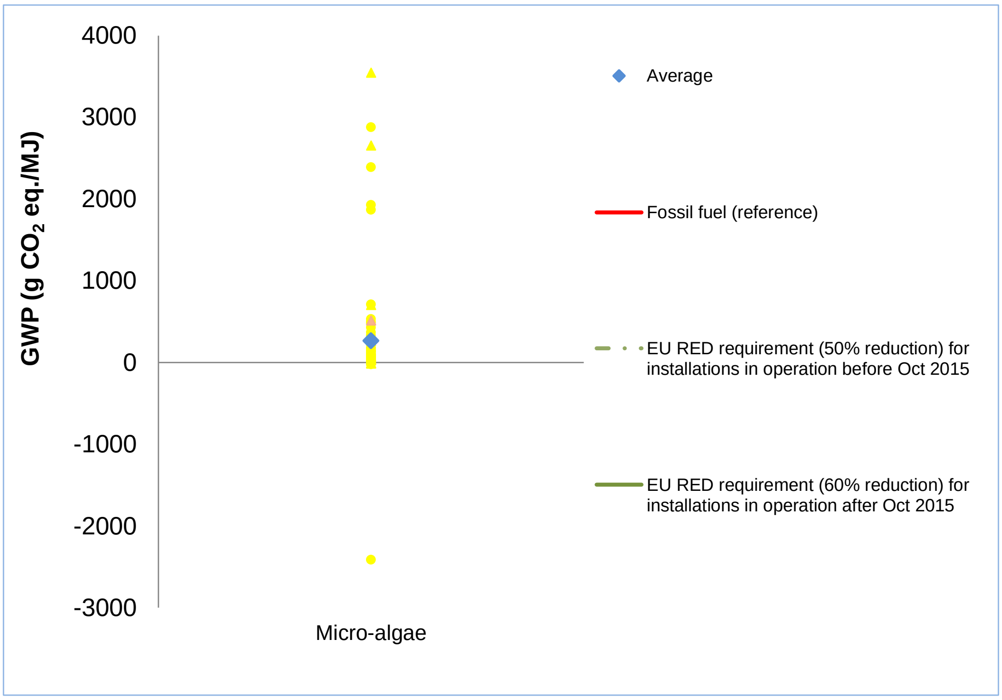

Figure S6: Global warming potential of algal biodiesel reported in LCA studies [27, 48, 70, 98, 131, 154-174]
[The values in this figure were used to generate plots in Figure 6 in the paper.]

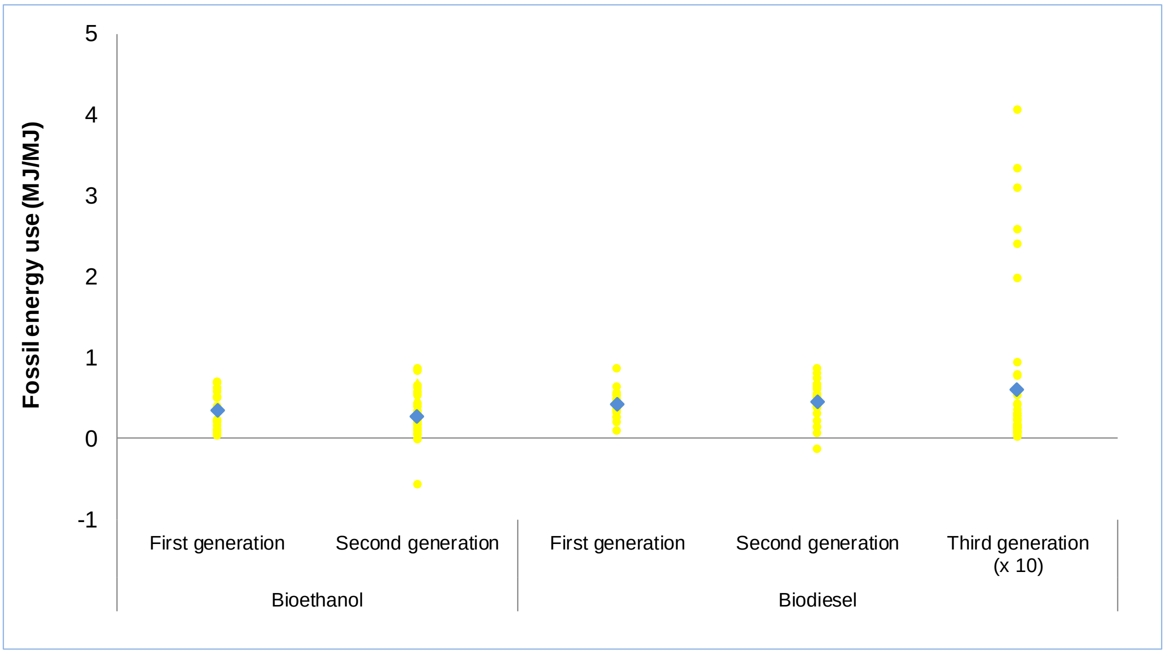

Figure S7: Fossil energy use in the life cycle of biofuels [1, 5, 8, 9, 12, 17, 18, 23, 27, 28, 33, 38, 41, 42, 44, 46, 47, 49, 50, 64, 70-72, 77, 83, 87, 88, 92, 94-96, 100, 101, 104, 105, 110-113, 115, 116, 119, 122, 123, 127, 129-131, 133-135, 139, 140, 143-145, 151, 154-162, 169, 171, 173, 175-179]

[The values in this figure were used to generate plots in Figure 7. The blue squares represent the average values across the studies. The values for 3rd generation biodiesel should be multiplied by 10 to obtain the actual value].

References

[1] Wang M, Han J, Dunn JB, Cai H, Elgowainy A. Well-to-wheels energy use and greenhouse gas emissions of ethanol from corn, sugarcane and cellulosic biomass for US use. Environ Res Lett. 2012;7.

[2] Canter CE, Dunn JB, Han J, Wang Z, Wang M. Policy Implications of Allocation Methods in the Life Cycle Analysis of Integrated Corn and Corn Stover Ethanol Production. Bioenergy Research. 2016;9:77-87.

[3] Muñoz I, Flury K, Jungbluth N, Rigarlsford G, i Canals LM, King H. Life cycle assessment of bio-based ethanol produced from different agricultural feedstocks. The International Journal of Life Cycle Assessment. 2014;19:109-19.

[4] Kim S, Dale BE. Regional variations in greenhouse gas emissions of biobased products in the United States—corn-based ethanol and soybean oil. The International Journal of Life Cycle Assessment. 2009;14:540-6.

[5] Ou X, Zhang X, Chang S, Guo Q. Energy consumption and GHG emissions of six biofuel pathways by LCA in (the) People's Republic of China. Applied Energy. 2009;86:S197-S208.

[6] Liska AJ, Yang HS, Bremer VR, Klopfenstein TJ, Walters DT, Erickson GE, et al. Improvements in Life Cycle Energy Efficiency and Greenhouse Gas Emissions of Corn-Ethanol. Journal of Industrial Ecology. 2009;13:58-74.

[7] Kauffman N, Hayes D, Brown R. A life cycle assessment of advanced biofuel production from a hectare of corn. Fuel. 2011;90:3306-14.

[8] Belboom S, Bodson B, Léonard A. Does the production of Belgian bioethanol fit with European requirements on GHG emissions? Case of wheat. Biomass and Bioenergy. 2015;74:58-65.

[9] Martinez-Hernandez E, Ibrahim MH, Leach M, Sinclair P, Campbell GM, Sadhukhan J. Environmental sustainability analysis of UK whole-wheat bioethanol and CHP systems. Biomass and Bioenergy. 2013;50:52-64.

[10] Yan X, Boies AM. Quantifying the uncertainties in life cycle greenhouse gas emissions for UK wheat ethanol. Environmental Research Letters. 2013;8.

[11] Styles D, Gibbons J, Williams AP, Dauber J, Stichnothe H, Urban B, et al. Consequential life cycle assessment of biogas, biofuel and biomass energy options within an arable crop rotation. Global Change Biology Bioenergy. 2015;7:1305-20.
[12] Buchspies B, Kaltschmitt M. Life cycle assessment of bioethanol from wheat and sugar beet discussing environmental impacts of multiple concepts of co-product processing in the context of the European Renewable Energy Directive. Biofuels. 2016;7:141-53.
[13] Gallejones P, Pardo G, Aizpurua A, del Prado A. Life cycle assessment of first-generation biofuels using a nitrogen crop model. Science of the Total Environment. 2015;505:1191-201.
[14] Elsgaard L, Olesen JE, Hermansen JE, Kristensen IT, Borgesen CD. Regional greenhouse gas emissions from cultivation of winter wheat and winter rapeseed for biofuels in Denmark. Acta Agriculturae Scandinavica Section B-Soil and Plant Science. 2013;63:219-30.
[15] Weinberg J, Kaltschmitt M. Greenhouse gas emissions from first generation ethanol derived from wheat and sugar beet in Germany – Analysis and comparison of advanced by-product utilization pathways. Applied Energy. 2013;102:131-9.
[16] Wang L, Quiceno R, Price C, Malpas R, Woods J. Economic and GHG emissions analyses for sugarcane ethanol in Brazil: Looking forward. Renewable and Sustainable Energy Reviews. 2014;40:571-82.
[17] Souza SP, Seabra JEA. Integrated production of sugarcane ethanol and soybean biodiesel: Environmental and economic implications of fossil diesel displacement. Energy Conversion and Management. 2014;87:1170-9.
[18] Tsiropoulos I, Faaij APC, Seabra JEA, Lundquist L, Schenker U, Briois J-F, et al. Life cycle assessment of sugarcane ethanol production in India in comparison to Brazil. International Journal of Life Cycle Assessment. 2014;19:1049-67.
[19] Cavalett O, Chagas MF, Seabra JEA, Bonomi A. Comparative LCA of ethanol versus gasoline in Brazil using different LCIA methods. The International Journal of Life Cycle Assessment. 2013;18:647-58.
[20] Mandade P, Bakshi BR, Yadav GD. Ethanol from Indian agro-industrial lignocellulosic biomass—a life cycle evaluation of energy, greenhouse gases, land and water. The International Journal of Life Cycle Assessment. 2015;20:1649-58.
[21] Tomaschek J, Oezdemir ED, Fahl U, Eltrop L. Greenhouse gas emissions and abatement costs of biofuel production in South Africa. Global Change Biology Bioenergy. 2012;4:799-810.
[22] Walter A, Dolzan P, Quilodrán O, de Oliveira JG, da Silva C, Piacente F, et al. Sustainability assessment of bio-ethanol production in Brazil considering land use change, GHG emissions and socio-economic aspects. Energy Policy. 2011;39:5703-16.
[23] García CA, Fuentes A, Hennecke A, Riegelhaupt E, Manzini F, Masera O. Life-cycle greenhouse gas emissions and energy balances of sugarcane ethanol production in Mexico. Applied Energy. 2011;88:2088-97.
[24] Seabra JEA, Macedo IC, Chum HL, Faroni CE, Sarto CA. Life cycle assessment of Brazilian sugarcane products: GHG emissions and energy use. Biofuels, Bioproducts and Biorefining. 2011;5:519-32.
[25] Bessou C, Lehuger S, Gabrielle B, Mary B. Using a crop model to account for the effects of local factors on the LCA of sugar beet ethanol in Picardy region, France. International Journal of Life Cycle Assessment. 2013;18:24-36.
[26] Halleux H, Lassaux S, Renzoni R, Germain A. Comparative life cycle assessment of two biofuels ethanol from sugar beet and rapeseed methyl ester. International Journal of Life Cycle Assessment. 2008;13:184-90.
[27] Souza SP, Gopal AR, Seabra JEA. Life cycle assessment of biofuels from an integrated Brazilian algae-sugarcane biorefinery. Energy. 2015;81:373-81.
[28] Michael W, Jeongwoo H, Jennifer BD, Hao C, Amgad E. Well-to-wheels energy use and greenhouse gas emissions of ethanol from corn, sugarcane and cellulosic biomass for US use. Environmental Research Letters. 2012;7:045905.
[29] Abdul-Manan AFN. Lifecycle GHG emissions of palm biodiesel: Unintended market effects negate direct benefits of the Malaysian Economic Transformation Plan (ETP). Energy Policy. 2017;104:56-65.

[30] Hassan MNA, Jaramillo P, Griffin WM. Life cycle GHG emissions from Malaysian oil palm bioenergy development: The impact on transportation sector's energy security. Energy Policy. 2011;39:2615-25.
[31] Harsono SS, Prochnow A, Grundmann P, Hansen A, Hallmann C. Energy balances and greenhouse gas emissions of palm oil biodiesel in Indonesia. GCB Bioenergy. 2012;4:213-28.
[32] Malca J, Coelho A, Freire F. Environmental life-cycle assessment of rapeseed-based biodiesel: Alternative cultivation systems and locations. Applied Energy. 2014;114:837-44.
[33] Stephenson AL, von Blottnitz H, Brent AC, Dennis JS, Scott SA. Global Warming Potential and Fossil-Energy Requirements of Biodiesel Production Scenarios in South Africa. Energy & Fuels. 2010;24:2489-99.
[34] Iriarte A, Rieradevall J, Gabarrell X. Transition towards a more environmentally sustainable biodiesel in South America: The case of Chile. Applied Energy. 2012;91:263-73.
[35] Arpornpong N, Sabatini DA, Khaodhiar S, Charoensaeng A. Life cycle assessment of palm oil microemulsion-based biofuel. International Journal of Life Cycle Assessment. 2015;20:913-26.
[36] Castanheira ÉG, Freire F. Environmental life cycle assessment of biodiesel produced with palm oil from Colombia. The International Journal of Life Cycle Assessment. 2017:587-600.
[37] Reinhard J, Zah R. Global environmental consequences of increased biodiesel consumption in Switzerland: consequential life cycle assessment. Journal of Cleaner Production. 2009;17, Supplement 1:S46-S56.
[38] Arvidsson R, Persson S, Froling M, Svanstrom M. Life cycle assessment of hydrotreated vegetable oil from rape, oil palm and Jatropha. Journal of Cleaner Production. 2011;19:129-37.
[39] Intarapong P, Papong S, Malakul P. Comparative life cycle assessment of diesel production from crude palm oil and waste cooking oil via pyrolysis. International Journal of Energy Research. 2016;40:702-13.
[40] Pehnelt G, Vietze C. Recalculating GHG emissions saving of palm oil biodiesel. Environment, Development and Sustainability. 2013;15:429-79.
[41] de Souza SP, Pacca S, de Ávila MT, Borges JLB. Greenhouse gas emissions and energy balance of palm oil biofuel. Renewable Energy. 2010;35:2552-61.
[42] Pleanjai S, Gheewala SH. Full chain energy analysis of biodiesel production from palm oil in Thailand. Applied Energy. 2009;86, Supplement 1:S209-S14.
[43] Ukaew S, Beck E, Archer DW, Shonnard DR. Estimation of soil carbon change from rotation cropping of rapeseed with wheat in the hydrotreated renewable jet life cycle. The International Journal of Life Cycle Assessment. 2015;20:608-22.
[44] Gonzalez-Garcia S, Garcia-Rey D, Hospido A. Environmental life cycle assessment for rapeseed-derived biodiesel. International Journal of Life Cycle Assessment. 2013;18:61-76.
[45] Sieverding HL, Bailey LM, Hengen TJ, Clay DE, Stone JJ. Meta-Analysis of Soybean-based Biodiesel. Journal of Environmental Quality. 2015;44:1038-48.
[46] Garrain D, Herrera I, Lechon Y, Lago C. Well-to-Tank environmental analysis of a renewable diesel fuel from vegetable oil through co-processing in a hydrotreatment unit. Biomass & Bioenergy. 2014;63:239-49.
[47] Kalnes TN, Koers KP, Marker T, Shonnard DR. A Technoeconomic and Environmental Life Cycle Comparison of Green Diesel to Biodiesel and Syndiesel. Environmental Progress & Sustainable Energy. 2009;28:111-20.
[48] Hou J, Zhang P, Yuan X, Zheng Y. Life cycle assessment of biodiesel from soybean, jatropha and microalgae in China conditions. Renewable & Sustainable Energy Reviews. 2011;15:5081-91.
[49] Iriarte A, Villalobos P. Greenhouse gas emissions and energy balance of sunflower biodiesel: Identification of its key factors in the supply chain. Resources Conservation and Recycling. 2013;73:46-52.
[50] Spinelli D, Jez S, Pogni R, Basosi R. Environmental and life cycle analysis of a biodiesel production line from sunflower in the Province of Siena (Italy). Energy Policy. 2013;59:492-506.
[51] Reijnders L, Huijbregts MAJ. Biogenic greenhouse gas emissions linked to the life cycles of biodiesel derived from European rapeseed and Brazilian soybeans. Journal of Cleaner Production. 2008;16:1943-8.
[52] Yee KF, Tan KT, Abdullah AZ, Lee KT. Life cycle assessment of palm biodiesel: Revealing facts and benefits for sustainability. Applied Energy. 2009;86, Supplement 1:S189-S96.

[53] Herrmann IT, Jørgensen A, Bruun S, Hauschild MZ. Potential for optimized production and use of rapeseed biodiesel. Based on a comprehensive real-time LCA case study in Denmark with multiple pathways. The International Journal of Life Cycle Assessment. 2013;18:418-30.
[54] Acquaye AA, Wiedmann T, Feng K, Crawford RH, Barrett J, Kuylenstierna J, et al. Identification of 'Carbon Hot-Spots' and Quantification of GHG Intensities in the Biodiesel Supply Chain Using Hybrid LCA and Structural Path Analysis. Environmental Science & Technology. 2011;45:2471-8.
[55] Buchspies B, Kaltschmitt M. A consequential assessment of changes in greenhouse gas emissions due to the introduction of wheat straw ethanol in the context of European legislation. Applied Energy. 2018;211:368-81.
[56] Klein BC, Chagas MF, Watanabe MDB, Bonomi A, Maciel Filho R. Low carbon biofuels and the New Brazilian National Biofuel Policy (RenovaBio): A case study for sugarcane mills and integrated sugarcane-microalgae biorefineries. Renewable and Sustainable Energy Reviews. 2019;115:109365.
[57] Maga D, Thonemann N, Hiebel M, Sebastião D, Lopes TF, Fonseca C, et al. Comparative life cycle assessment of first- and second-generation ethanol from sugarcane in Brazil. The International Journal of Life Cycle Assessment. 2019;24:266-80.
[58] Vera I, Hoefnagels R, van der Kooij A, Moretti C, Junginger M. A carbon footprint assessment of multi-output biorefineries with international biomass supply: a case study for the Netherlands. Biofuels, Bioproducts and Biorefining. 2020;14:198-224.
[59] Alexiades A, Kendall A, Winans KS, Kaffka SR. Sugar beet ethanol (Beta vulgaris L.): A promising low-carbon pathway for ethanol production in California. Journal of Cleaner Production. 2018;172:3907-17.
[60] Meijide A, de la Rua C, Guillaume T, Röll A, Hassler E, Stiegler C, et al. Measured greenhouse gas budgets challenge emission savings from palm-oil biodiesel. Nature Communications. 2020;11:1089.
[61] Prapaspongsa T, Gheewala SH. Consequential and attributional environmental assessment of biofuels: implications of modelling choices on climate change mitigation strategies. The International Journal of Life Cycle Assessment. 2017;22:1644-57.
[62] Prapaspongsa T, Musikavong C, Gheewala SH. Life cycle assessment of palm biodiesel production in Thailand: Impacts from modelling choices, co-product utilisation, improvement technologies, and land use change. Journal of Cleaner Production. 2017;153:435-47.
[63] Ratthanaphra D, Suwanmanee U. Uncertainty analysis of environmental sustainability of biodiesel production using Thai domestic rare earth oxide solid catalysts. Sustainable Production and Consumption. 2019;18:237-49.
[64] Chen R, Qin Z, Han J, Wang M, Taheripour F, Tyner W, et al. Life cycle energy and greenhouse gas emission effects of biodiesel in the United States with induced land use change impacts. Bioresource Technology. 2018;251:249-58.
[65] O'Keeffe S, Majer S, Drache C, Franko U, Thrän D. Modelling biodiesel production within a regional context – A comparison with RED Benchmark. Renewable Energy. 2017;108:355-70.
[66] Knoope MMJ, Balzer CH, Worrell E. Analysing the water and greenhouse gas effects of soya bean-based biodiesel in five different regions. GCB Bioenergy. 2019;11:381-99.
[67] Cerri CEP, You X, Cherubin MR, Moreira CS, Raucci GS, Castigioni BdA, et al. Assessing the greenhouse gas emissions of Brazilian soybean biodiesel production. PLOS ONE. 2017;12:e0176948.
[68] Esteves EMM, Esteves VPP, Bungenstab DJ, Araújo OdQF, Morgado CdRV. Greenhouse gas emissions related to biodiesel from traditional soybean farming compared to integrated crop-livestock systems. Journal of Cleaner Production. 2018;179:81-92.
[69] Renouf MA, Pagan RJ, Wegener MK. Life cycle assessment of Australian sugarcane products with a focus on cane processing. The International Journal of Life Cycle Assessment. 2011;16:125-37.
[70] Cox K, Renouf M, Dargan A, Turner C, Klein-Marcuschamer D. Environmental life cycle assessment (LCA) of aviation biofuel from microalgae, Pongamia pinnata, and sugarcane molasses. Biofuels Bioproducts & Biorefining-Biofpr. 2014;8:579-93.

[71] Gabisa EW, Bessou C, Gheewala SH. Life cycle environmental performance and energy balance of ethanol production based on sugarcane molasses in Ethiopia. Journal of Cleaner Production. 2019;234:43-53.
[72] Rathnayake M, Chaireongsirikul T, Svangariyaskul A, Lawtrakul L, Toochinda P. Process simulation based life cycle assessment for bioethanol production from cassava, cane molasses, and rice straw. Journal of Cleaner Production. 2018;190:24-35.
[73] Papong S, Rewlay-ngoen C, Itsubo N, Malakul P. Environmental life cycle assessment and social impacts of bioethanol production in Thailand. Journal of Cleaner Production. 2017;157:254-66.
[74] Silalertruksa T, Gheewala SH. A comparative LCA of rice straw utilization for fuels and fertilizer in Thailand. Bioresource Technology. 2013;150:412-9.
[75] Khatiwada D, Silveira S. Greenhouse gas balances of molasses based ethanol in Nepal. Journal of Cleaner Production. 2011;19:1471-85.
[76] Soam S, Kumar R, Gupta RP, Sharma PK, Tuli DK, Das B. Life cycle assessment of fuel ethanol from sugarcane molasses in northern and western India and its impact on Indian biofuel programme. Energy. 2015;83:307-15.
[77] Zhang Y, Kendall A. Life Cycle Performance of Cellulosic Ethanol and Corn Ethanol from a Retrofitted Dry Mill Corn Ethanol Plant. Bioenergy Research. 2017:183-98.
[78] Dunn JB, Mueller S, Kwon H-y, Wang MQ. Land-use change and greenhouse gas emissions from corn and cellulosic ethanol. Biotechnology for Biofuels. 2013;6:51.
[79] Reijnders L, Huijbregts MAJ. Palm oil and the emission of carbon-based greenhouse gases. Journal of Cleaner Production. 2008;16:477-82.
[80] Harding KG, Dennis JS, von Blottnitz H, Harrison STL. A life-cycle comparison between inorganic and biological catalysis for the production of biodiesel. Journal of Cleaner Production. 2008;16:1368-78.
[81] Fernández-Tirado F, Parra-López C, Romero-Gámez M. Life cycle assessment of biodiesel in Spain: Comparing the environmental sustainability of Spanish production versus Argentinean imports. Energy for Sustainable Development. 2016;33:36-52.
[82] Tonini D, Astrup T. LCA of biomass-based energy systems: A case study for Denmark. Applied Energy. 2012;99:234-46.
[83] Panichelli L, Dauriat A, Gnansounou E. Life cycle assessment of soybean-based biodiesel in Argentina for export. International Journal of Life Cycle Assessment. 2009;14:144-59.
[84] Hansen S. Feasibility Study of Performing an Life Cycle Assessment on Crude Palm Oil Production in Malaysia (9 pp). The International Journal of Life Cycle Assessment. 2007;12:50-8.
[85] Kesieme U, Pazouki K, Murphy A, Chrysanthou A. Attributional life cycle assessment of biofuels for shipping: Addressing alternative geographical locations and cultivation systems. Journal of Environmental Management. 2019;235:96-104.
[86] Tonini D, Hamelin L, Alvarado-Morales M, Astrup TF. GHG emission factors for bioelectricity, biomethane, and bioethanol quantified for 24 biomass substrates with consequential life-cycle assessment. Bioresource Technology. 2016;208:123-33.
[87] Jeswani HK, Falano T, Azapagic A. Life cycle environmental sustainability of lignocellulosic ethanol produced in integrated thermo-chemical biorefineries. Biofuels Bioproducts & Biorefining-Biofpr. 2015;9:661-76.
[88] Budsberg E, Crawford J, Gustafson R, Bura R, Puettmann M. Ethanologens vs. acetogens: Environmental impacts of two ethanol fermentation pathways. Biomass & Bioenergy. 2015;83:23-31.
[89] Brynolf S, Fridell E, Andersson K. Environmental assessment of marine fuels: liquefied natural gas, liquefied biogas, methanol and bio-methanol. Journal of Cleaner Production. 2014;74:86-95.
[90] Guo M, Littlewood J, Joyce J, Murphy R. The environmental profile of bioethanol produced from current and potential future poplar feedstocks in the EU. Green Chemistry. 2014;16:4680-95.
[91] Guo M, Li C, Facciotto G, Bergante S, Bhatia R, Comolli R, et al. Bioethanol from poplar clone Imola: an environmentally viable alternative to fossil fuel? Biotechnology for Biofuels. 2015;8.
[92] Falano T, Jeswani HK, Azapagic A. Assessing the environmental sustainability of ethanol from integrated biorefineries. Biotechnology Journal. 2014;9:753-65.

[93] Reyes Valle C, Villanueva Perales AL, Vidal-Barrero F, Ollero P. Integrated economic and life cycle assessment of thermochemical production of bioethanol to reduce production cost by exploiting excess of greenhouse gas savings. Applied Energy. 2015;148:466-75.
[94] Stephenson AL, Dupree P, Scott SA, Dennis JS. The environmental and economic sustainability of potential bioethanol from willow in the UK. Bioresource Technology. 2010;101:9612-23.
[95] Iribarren D, Peters JF, Dufour J. Life cycle assessment of transportation fuels from biomass pyrolysis. Fuel. 2012;97:812-21.
[96] González-García S, Iribarren D, Susmozas A, Dufour J, Murphy RJ. Life cycle assessment of two alternative bioenergy systems involving Salix spp. biomass: Bioethanol production and power generation. Applied Energy. 2012;95:111-22.
[97] Weinberg J, Kaltschmitt M. Life cycle assessment of mobility options using wood based fuels - Comparison of selected environmental effects and costs. Bioresource Technology. 2013;150:420-8.
[98] Maleche E, Glaser R, Marker T, Shonnard D. A Preliminary Life Cycle Assessment of Biofuels Produced by the IH2 (TM) Process. Environmental Progress & Sustainable Energy. 2014;33:322-9.
[99] Slade R, Bauen A, Shah N. The greenhouse gas emissions performance of cellulosic ethanol supply chains in Europe. Biotechnology for Biofuels. 2009;2:15-.
[100] Daystar J, Reeb C, Gonzalez R, Venditti R, Kelley SS. Environmental life cycle impacts of cellulosic ethanol in the Southern US produced from loblolly pine, eucalyptus, unmanaged hardwoods, forest residues, and switchgrass using a thermochemical conversion pathway. Fuel Processing Technology. 2015;138:164-74.
[101] Zaimes GG, Soratana K, Harden CL, Landis AE, Khanna V. Biofuels via Fast Pyrolysis of Perennial Grasses: A Life Cycle Evaluation of Energy Consumption and Greenhouse Gas Emissions. Environmental Science & Technology. 2015;49:10007-18.
[102] Scown CD, Nazaroff WW, Mishra U, Strogen B, Lobscheid AB, Masanet E, et al. Lifecycle greenhouse gas implications of US national scenarios for cellulosic ethanol production. Environ Res Lett. 2012;7.
[103] Choudhary S, Liang S, Cai H, Keoleian GA, Miller SA, Kelly J, et al. Reference and functional unit can change bioenergy pathway choices. The International Journal of Life Cycle Assessment. 2014;19:796-805.
[104] Sinistore JC, Reinemann DJ, Izaurralde RC, Cronin KR, Meier PJ, Runge TM, et al. Life Cycle Assessment of Switchgrass Cellulosic Ethanol Production in the Wisconsin and Michigan Agricultural Contexts. Bioenergy Research. 2015;8:897-909.
[105] Cherubini F, Jungmeier G. LCA of a biorefinery concept producing bioethanol, bioenergy, and chemicals from switchgrass. The International Journal of Life Cycle Assessment. 2010;15:53-66.
[106] Bai Y, Luo L, van der Voet E. Life cycle assessment of switchgrass-derived ethanol as transport fuel. The International Journal of Life Cycle Assessment. 2010;15:468-77.
[107] Argo AM, Tan ECD, Inman D, Langholtz MH, Eaton LM, Jacobson JJ, et al. Investigation of biochemical biorefinery sizing and environmental sustainability impacts for conventional bale system and advanced uniform biomass logistics designs. Biofuels Bioproducts & Biorefining-Biofpr. 2013;7:282-302.
[108] Patrizi N, Caro D, Pulselli FM, Bjerre AB, Bastianoni S. Environmental feasibility of partial substitution of gasoline with ethanol in the Province of Siena (Italy). Journal of Cleaner Production. 2013;47:388-95.
[109] Moller F, Slento E, Frederiksen P. Integrated well-to-wheel assessment of biofuels combining energy and emission LCA and welfare economic Cost Benefit Analysis. Biomass & Bioenergy. 2014;60:41-9.
[110] Peters JF, Iribarren D, Dufour J. Simulation and life cycle assessment of biofuel production via fast pyrolysis and hydroupgrading. Fuel. 2015;139:441-56.
[111] Kumar D, Murthy GS. Life cycle assessment of energy and GHG emissions during ethanol production from grass straws using various pretreatment processes. The International Journal of Life Cycle Assessment. 2012;17:388-401.
[112] Wang L, Littlewood J, Murphy RJ. Environmental sustainability of bioethanol production from wheat straw in the UK. Renewable & Sustainable Energy Reviews. 2013;28:715-25.

[113] Dang Q, Yu C, Luo Z. Environmental life cycle assessment of bio-fuel production via fast pyrolysis of corn stover and hydroprocessing. Fuel. 2014;131:36-42.
[114] Nguyen L, Cafferty KG, Searcy EM, Spatari S. Uncertainties in Life Cycle Greenhouse Gas Emissions from Advanced Biomass Feedstock Logistics Supply Chains in Kansas. Energies. 2014;7:7125-46.
[115] Palma-Rojas S, Caldeira-Pires A, Nogueira JM. Environmental and economic hybrid life cycle assessment of bagasse-derived ethanol produced in Brazil. The International Journal of Life Cycle Assessment. 2017;22:317-27.
[116] Daystar J, Treasure T, Gonzalez R, Reeb C, Venditti R, Kelley S. The NREL Biochemical and Thermochemical Ethanol Conversion Processes: Financial and Environmental Analysis Comparison. Bioresources. 2015;10:5096-116.
[117] Wang H, Zhang S, Bi X, Clift R. Greenhouse gas emission reduction potential and cost of bioenergy in British Columbia, Canada. Energy Policy. 2020;138:111285.
[118] Haus S, Björnsson L, Börjesson P. Lignocellulosic Ethanol in a Greenhouse Gas Emission Reduction Obligation System—A Case Study of Swedish Sawdust Based-Ethanol Production. Energies. 2020;13:1048.
[119] Zupko R. Life cycle assessment of the production of gasoline and diesel from forest residues using integrated hydropyrolysis and hydroconversion. The International Journal of Life Cycle Assessment. 2019;24:1793-804.
[120] Lask J, Wagner M, Trindade LM, Lewandowski I. Life cycle assessment of ethanol production from miscanthus: A comparison of production pathways at two European sites. GCB Bioenergy. 2019;11:269-88.
[121] Sreekumar A, Shastri Y, Wadekar P, Patil M, Lali A. Life cycle assessment of ethanol production in a rice-straw-based biorefinery in India. Clean Technologies and Environmental Policy. 2020;22:409-22.
[122] Gonzalez-Garcia S, Teresa Moreira M, Feijoo G. Comparative environmental performance of lignocellulosic ethanol from different feedstocks. Renewable & Sustainable Energy Reviews. 2010;14:2077-85.
[123] Parajuli R, Knudsen MT, Birkved M, Djomo SN, Corona A, Dalgaard T. Environmental impacts of producing bioethanol and biobased lactic acid from standalone and integrated biorefineries using a consequential and an attributional life cycle assessment approach. Science of The Total Environment. 2017;598:497-512.
[124] Zhao Y, Damgaard A, Xu Y, Liu S, Christensen TH. Bioethanol from corn stover – Global warming footprint of alternative biotechnologies. Applied Energy. 2019;247:237-53.
[125] Obnamia JA, Dias GM, MacLean HL, Saville BA. Comparison of U.S. Midwest corn stover ethanol greenhouse gas emissions from GREET and GHGenius. Applied Energy. 2019;235:591-601.
[126] Liu C, Huang Y, Wang X, Tai Y, Liu L, Liu H. Total environmental impacts of biofuels from corn stover using a hybrid life cycle assessment model combining process life cycle assessment and economic input–output life cycle assessment. Integrated Environmental Assessment and Management. 2018;14:139-49.
[127] Eshton B, Katima JHY, Kituyi E. Greenhouse gas emissions and energy balances of jatropha biodiesel as an alternative fuel in Tanzania. Biomass & Bioenergy. 2013;58:95-103.
[128] Shonnard DR, Klemetsrud B, Sacramento-Rivero J, Navarro-Pineda F, Hilbert J, Handler R, et al. A Review of Environmental Life Cycle Assessments of Liquid Transportation Biofuels in the Pan American Region. Environmental Management. 2015;56:1356-76.
[129] Hagman J, Nerentorp M, Arvidsson R, Molander S. Do biofuels require more water than do fossil fuels? Life cycle-based assessment of jatropha oil production in rural Mozambique. Journal of Cleaner Production. 2013;53:176-85.
[130] Ndong R, Montrejaud-Vignoles M, Saint Girons O, Gabrielle B, Pirot R, Domergue M, et al. Life cycle assessment of biofuels from Jatropha curcas in West Africa: a field study. GCB Bioenergy. 2009;1:197-210.
[131] Ajayebi A, Gnansounou E, Raman JK. Comparative life cycle assessment of biodiesel from algae and jatropha: A case study of India. Bioresource Technology. 2013;150:429-37.
[132] Kumar S, Singh J, Nanoti SM, Garg MO. A comprehensive life cycle assessment (LCA) of Jatropha biodiesel production in India. Bioresource Technology. 2012;110:723-9.

[133] Li X, Mupondwa E. Life cycle assessment of camelina oil derived biodiesel and jet fuel in the Canadian Prairies. Science of the Total Environment. 2014;481:17-26.
[134] Krohn BJ, Fripp M. A life cycle assessment of biodiesel derived from the "niche filling" energy crop camelina in the USA. Applied Energy. 2012;92:92-8.
[135] Escobar N, Ribal J, Clemente G, Sanjuan N. Consequential LCA of two alternative systems for biodiesel consumption in Spain, considering uncertainty. Journal of Cleaner Production. 2014;79:61-73.
[136] Caldeira C, Queirós J, Noshadravan A, Freire F. Incorporating uncertainty in the life cycle assessment of biodiesel from waste cooking oil addressing different collection systems. Resources, Conservation and Recycling. 2016;112:83-92.
[137] Talens Peiró L, Lombardi L, Villalba Méndez G, Gabarrell i Durany X. Life cycle assessment (LCA) and exergetic life cycle assessment (ELCA) of the production of biodiesel from used cooking oil (UCO). Energy. 2010;35:889-93.
[138] Dufour J, Iribarren D. Life cycle assessment of biodiesel production from free fatty acid-rich wastes. Renewable Energy. 2012;38:155-62.
[139] Thamsiriroj T, Murphy JD. The impact of the life cycle analysis methodology on whether biodiesel produced from residues can meet the EU sustainability criteria for biofuel facilities constructed after 2017. Renewable Energy. 2011;36:50-63.
[140] Souza DdP, Mendonca FM, Alves Nunes KR, Valle R. Environmental and Socioeconomic Analysis of Producing Biodiesel from Used Cooking Oil in Rio de Janeiro The Case of the Copacabana District. Journal of Industrial Ecology. 2012;16:655-64.
[141] Pleanjai S, Gheewala SH, Garivait S. Greenhouse gas emissions from production and use of used cooking oil methyl ester as transport fuel in Thailand. Journal of Cleaner Production. 2009;17:873-6.
[142] Mortimer N, Evans AKF, Mwabonje O, Whittaker CL, Hunter AJ. Comparison of the Greenhouse Gas Benefits Resulting from Use of Vegetable Oils for Electricty, Heat, Transport and Industrial Purposes. NNFCC; 2010.
[143] Vrech A, Ferfuia C, Bessong Ojong W, Piasentier E, Baldini M. Energy and environmental sustainability of Jatropha-Biofuels Chain from nontoxic accessions in Cameroon. Environmental Progress & Sustainable Energy. 2019;38:305-14.
[144] Fuentes A, García C, Hennecke A, Masera O. Life cycle assessment of Jatropha curcas biodiesel production: a case study in Mexico. Clean Technologies and Environmental Policy. 2018;20:1721-33.
[145] Baumert S, Khamzina A, Vlek PLG. Greenhouse gas and energy balance of Jatropha biofuel production systems of Burkina Faso. Energy for Sustainable Development. 2018;42:14-23.
[146] Khang DS, Tan RR, Uy OM, Promentilla MAB, Tuan PD, Abe N, et al. Design of experiments for global sensitivity analysis in life cycle assessment: The case of biodiesel in Vietnam. Resources, Conservation and Recycling. 2017;119:12-23.
[147] Giraldi-Díaz MR, De Medina-Salas L, Castillo-González E, De la Cruz-Benavides M. Environmental Impact Associated with the Supply Chain and Production of Biodiesel from Jatropha curcas L. through Life Cycle Analysis. Sustainability. 2018;10:1451.
[148] Bacenetti J, Restuccia A, Schillaci G, Failla S. Biodiesel production from unconventional oilseed crops (Linum usitatissimum L. and Camelina sativa L.) in Mediterranean conditions: Environmental sustainability assessment. Renewable Energy. 2017;112:444-56.
[149] Tabatabaie SMH, Tahami H, Murthy GS. A regional life cycle assessment and economic analysis of camelina biodiesel production in the Pacific Northwestern US. Journal of Cleaner Production. 2018;172:2389-400.
[150] Foteinis S, Chatzisymeon E, Litinas A, Tsoutsos T. Used-cooking-oil biodiesel: Life cycle assessment and comparison with first- and third-generation biofuel. Renewable Energy. 2020;153:588-600.
[151] Faleh N, Khila Z, Wahada Z, Pons M-N, Houas A, Hajjaji N. Exergo-environmental life cycle assessment of biodiesel production from mutton tallow transesterification. Renewable Energy. 2018;127:74-83.
[152] Lombardi L, Mendecka B, Carnevale E. Comparative life cycle assessment of alternative strategies for energy recovery from used cooking oil. Journal of Environmental Management. 2018;216:235-45.

[153] Yang Y, Fu T, Bao W, Xie GH. Life Cycle Analysis of Greenhouse Gas and PM2.5 Emissions from Restaurant Waste Oil Used for Biodiesel Production in China. BioEnergy Research. 2017;10:199-207.
[154] Pragya N, Pandey KK. Life cycle assessment of green diesel production from microalgae. Renewable Energy. 2016;86:623-32.
[155] Medeiros DL, Sales EA, Kiperstok A. Energy production from microalgae biomass: carbon footprint and energy balance. Journal of Cleaner Production. 2015;96:493-500.
[156] Adesanya VO, Cadena E, Scott SA, Smith AG. Life cycle assessment on microalgal biodiesel production using a hybrid cultivation system. Bioresource Technology. 2014;163:343-55.
[157] Passell H, Dhaliwal H, Reno M, Wu B, Ben Amotz A, Ivry E, et al. Algae biodiesel life cycle assessment using current commercial data. Journal of Environmental Management. 2013;129:103-11.
[158] Yuan J, Kendall A, Zhang Y. Mass balance and life cycle assessment of biodiesel from microalgae incorporated with nutrient recycling options and technology uncertainties. Global Change Biology Bioenergy. 2015;7:1245-59.
[159] Mu D, Min M, Krohn B, Mullins KA, Ruan R, Hill J. Life Cycle Environmental Impacts of Wastewater-Based Algal Biofuels. Environmental Science & Technology. 2014;48:11696-704.
[160] Sills DL, Paramita V, Franke MJ, Johnson MC, Akabas TM, Greene CH, et al. Quantitative Uncertainty Analysis of Life Cycle Assessment for Algal Biofuel Production. Environmental Science & Technology. 2013;47:687-94.
[161] Campbell PK, Beer T, Batten D. Life cycle assessment of biodiesel production from microalgae in ponds. Bioresource Technology. 2011;102:50-6.
[162] Stephenson AL, Kazamia E, Dennis JS, Howe CJ, Scott SA, Smith AG. Life-Cycle Assessment of Potential Algal Biodiesel Production in the United Kingdom: A Comparison of Raceways and Air-Lift Tubular Bioreactors. Energy & Fuels. 2010;24:4062-77.
[163] Sander K, Murthy GS. Life cycle analysis of algae biodiesel. The International Journal of Life Cycle Assessment. 2010;15:704-14.
[164] Holma A, Koponen K, Antikainen R, Lardon L, Leskinen P, Roux P. Current limits of life cycle assessment framework in evaluating environmental sustainability - case of two evolving biofuel technologies. Journal of Cleaner Production. 2013;54:215-28.
[165] Woertz IC, Benemann JR, Du N, Unnasch S, Mendola D, Mitchell BG, et al. Life Cycle GHG Emissions from Microalgal Biodiesel - A CA-GREET Model. Environmental Science & Technology. 2014;48:6060-8.
[166] Soratana K, Harper WF, Jr., Landis AE. Microalgal biodiesel and the Renewable Fuel Standard's greenhouse gas requirement. Energy Policy. 2012;46:498-510.
[167] Chowdhurya R, Freire F. Bioenergy production from algae using dairy manure as a nutrient source: Life cycle energy and greenhouse gas emission analysis. Applied Energy. 2015;154:1112-21.
[168] Bennion EP, Ginosar DM, Moses J, Agblevor F, Quinn JC. Lifecycle assessment of microalgae to biofuel: Comparison of thermochemical processing pathways. Applied Energy. 2015;154:1062-71.
[169] Azari A, Noorpoor AR, Bozorg-Haddad O. Carbon footprint analyses of microalgae cultivation systems under autotrophic and heterotrophic conditions. International Journal of Environmental Science and Technology. 2019;16:6671-84.
[170] Morales M, Hélias A, Bernard O. Optimal integration of microalgae production with photovoltaic panels: environmental impacts and energy balance. Biotechnology for Biofuels. 2019;12:239.
[171] Sun C-H, Fu Q, Liao Q, Xia A, Huang Y, Zhu X, et al. Life-cycle assessment of biofuel production from microalgae via various bioenergy conversion systems. Energy. 2019;171:1033-45.
[172] Wu W, Lin K-H, Chang J-S. Economic and life-cycle greenhouse gas optimization of microalgae-to-biofuels chains. Bioresource Technology. 2018;267:550-9.
[173] Foteinis S, Antoniadis-Gavriil A, Tsoutsos T. Life cycle assessment of algae-to-biodiesel shallow pond production systems in the Mediterranean: influence of species, pond type, by(co)-product valorisation and electricity mix. Biofuels, Bioproducts and Biorefining. 2018;12:542-58.

[174] Bello M, Ranganathan P, Brennan F. Life Cycle Optimization for Sustainable Algal Biofuel Production Using Integrated Nutrient Recycling Technology. ACS Sustainable Chemistry & Engineering. 2017;5:9869-80.
[175] Ponnusamy S, Reddy HK, Muppaneni T, Downes CM, Deng S. Life cycle assessment of biodiesel production from algal bio-crude oils extracted under subcritical water conditions. Bioresource Technology. 2014;170:454-61.
[176] Pardo-Cardenas Y, Herrera-Orozco I, Gonzalez-Delgado A-D, Kafarov V. Environmental assessment of microalgae biodiesel production in Colombia: comparison of three oil extraction systems. Ct&F-Ciencia Tecnologia Y Futuro. 2013;5:85-100.
[177] Scacchi CCO, Gonzalez-Garcia S, Caserini S, Rigamonti L. Greenhouse gases emissions and energy use of wheat grain-based bioethanol fuel blends. Science of the Total Environment. 2010;408:5010-8.
[178] Kamahara H, Hasanudin U, Widiyanto A, Tachibana R, Atsuta Y, Goto N, et al. Improvement potential for net energy balance of biodiesel derived from palm oil: A case study from Indonesian practice. Biomass and Bioenergy. 2010;34:1818-24.
[179] Queiroz AG, Franca L, Ponte MX. The life cycle assessment of biodiesel from palm oil ("dende") in the Amazon. Biomass & Bioenergy. 2012;36:50-9.
